# *Homeobox B13* G84E Mutation and Prostate Cancer Risk

**DOI:** 10.1016/j.eururo.2018.11.015

**Published:** 2019-05

**Authors:** Tommy Nyberg, Koveela Govindasami, Goska Leslie, Tokhir Dadaev, Elizabeth Bancroft, Holly Ni Raghallaigh, Mark N. Brook, Nafisa Hussain, Diana Keating, Andrew Lee, Romayne McMahon, Angela Morgan, Andrea Mullen, Andrea Osborne, Reshma Rageevakumar, The UK Genetic Prostate Cancer Study Collaborators, Zsofia Kote-Jarai, Rosalind Eeles, Antonis C. Antoniou

**Affiliations:** a Centre for Cancer Genetic Epidemiology, Department of Public Health and Primary Care, University of Cambridge, Cambridge, UK; b Oncogenetics Team, Division of Cancer Genetics and Epidemiology, The Institute of Cancer Research, London, UK; c Royal Marsden NHS Foundation Trust, London, UK

**Keywords:** Genetic risk, *HomeoboxB13*, Kin-cohort study, Meta-analysis, Prostate cancer

## Abstract

**Background:**

The *homeobox B13* (*HOXB13*) G84E mutation has been recommended for use in genetic counselling for prostate cancer (PCa), but the magnitude of PCa risk conferred by this mutation is uncertain.

**Objective:**

To obtain precise risk estimates for mutation carriers and information on how these vary by family history and other factors.

**Design, setting, and participants:**

Two-fold: a systematic review and meta-analysis of published risk estimates, and a kin-cohort study comprising pedigree data on 11 983 PCa patients enrolled during 1993–2014 from 189 UK hospitals and who had been genotyped for *HOXB13* G84E.

**Outcome measurements and statistical analysis:**

Relative and absolute PCa risks. Complex segregation analysis with ascertainment adjustment to derive age-specific risks applicable to the population, and to investigate how these vary by family history and birth cohort.

**Results and limitations:**

A meta-analysis of case-control studies revealed significant heterogeneity between reported relative risks (RRs; range: 0.95–33.0, *p* < 0.001) and differences by case selection (*p* = 0.007). Based on case-control studies unselected for PCa family history, the pooled RR estimate was 3.43 (95% confidence interval [CI] 2.78–4.23). In the kin-cohort study, PCa risk for mutation carriers varied by family history (*p* < 0.001). There was a suggestion that RRs decrease with age, but this was not significant (*p* = 0.068). We found higher RR estimates for men from more recent birth cohorts (*p* = 0.004): 3.09 (95% CI 2.03–4.71) for men born in 1929 or earlier and 5.96 (95% CI 4.01–8.88) for men born in 1930 or later. The absolute PCa risk by age 85 for a male *HOXB13* G84E carrier varied from 60% for those with no PCa family history to 98% for those with two relatives diagnosed at young ages, compared with an average risk of 15% for noncarriers. Limitations include the reliance on self-reported cancer family history.

**Conclusions:**

PCa risks for *HOXB13* G84E mutation carriers are heterogeneous. Counselling should not be based on average risk estimates but on age-specific absolute risk estimates tailored to individual mutation carriers’ family history and birth cohort.

**Patient summary:**

Men who carry a hereditary mutation in the *homeobox B13* (*HOXB13*) gene have a higher than average risk for developing prostate cancer. In our study, we examined a large number of families of men with prostate cancer recruited across UK hospitals, to assess what other factors may contribute to this risk and to assess whether we could create a precise model to help in predicting a man's prostate cancer risk. We found that the risk of developing prostate cancer in men who carry this genetic mutation is also affected by a family history of prostate cancer and their year of birth. This information can be used to assess more personalised prostate cancer risks to men who carry *HOXB13* mutations and hence better counsel them on more personalised risk management options, such as tailoring prostate cancer screening frequency.

## Take Home Message

Men with a G84E mutation in the *HOXB13* gene are at a high risk of developing prostate cancer. The risk is however heterogeneous, and is modified by both family history and birth cohort. Genetic counselling should account for this heterogeneity.

## 1 Introduction

The *homeobox B13* (*HOXB13*) gene is involved in prostate development [1], and in vitro results have suggested that its transcription factor is involved in prostate cancer (PCa) cell growth through androgen receptor interaction, regulation by *FOXA1*, and other pathways [2]. The *HOXB13* missense mutation G84E is a founder mutation in Nordic populations [3], with reported carrier frequencies of 0.2–1.4% [4], [5], [6], [7], and is carried by 0.1–0.5% in other Western European populations [4], [8]. Mutation carrier frequencies are lower in Southern European populations [4], [9], and the variant is very rare in African and Asian ancestry populations [4], [9], [10]. *HOXB13* G84E is associated with PCa risk, but reported relative risks (RRs) have shown considerable heterogeneity and often wide confidence intervals (CIs) [3], [4], [5], [6], [7], [8], [9], [10], [11], [12], [13], [14], [15], [16], [17], [18], [19], [20], [21], [22]. Most risk estimates come from case-control studies, but because G84E mutations are rare, the small number of mutations in controls can lead to imprecision. Moreover, estimates may be biased if participants are not randomly recruited from cases unselected for age at diagnosis or family history, and if population-matched controls are not available. In contrast, kin-cohort or family-based studies, in which affected individuals are screened for the mutation and data on relatives are used to estimate cancer risks, enable observation of a larger number of mutation carriers, and often provide greater precision and unbiased estimates provided analyses are adjusted for ascertainment [23], [24], [25].

Genetic counselling for men at an elevated PCa risk has relied predominantly on family history, ethnicity [26], and *BRCA1* or *BRCA2* mutation carrier status [27]. Recently, a consensus conference recommended additionally testing for *HOXB13* mutations [28]. However, to provide individualised counselling to mutation carriers, it is important to have valid and precise age-specific cancer risk estimates and information on how other factors including PCa family history modify these risks.

The aim of the present analysis was two-fold. First, we performed a systematic review and meta-analysis of the PCa risk for *HOXB13* G84E carriers based on case selection and study ascertainment criteria. Previous meta-analyses [29], [30] combined RR estimates from unselected and high-risk cases, resulting in pooled estimates that may not be widely applicable. Second, using family data from the largest kin-cohort PCa study to date in which participants were genotyped for *HOXB13* G84E, we estimated relative and absolute PCa risks for mutation carriers, and assessed how PCa risks vary by family history, birth cohort, and age. We used the results to obtain clinically relevant absolute risk estimates by various PCa family history configurations, applicable to mutation carriers identified in different contexts, for example, in clinical genetics or through population-based screening programmes.

## 2 Patients and methods

### 2.1 Systematic review and meta-analysis

We performed a systematic review and meta-analysis to synthesise the available evidence on *HOXB13* G84E mutation and PCa risk. Details are given in the Supplementary material (systematic review and meta-analysis).

### 2.2 Risk estimation

#### 2.2.1 Study participants: the United Kingdom Genetic Prostate Cancer Study

Between January 1993 and November 2014, men diagnosed with histologically confirmed PCa at one of 189 UK hospitals were recruited into the three arms of the United Kingdom Genetic Prostate Cancer Study (UKGPCS). The population-based *PRM* arm invited all men diagnosed or treated at the Royal Marsden NHS Foundation Trust. The young-onset *PRY* arm invited men diagnosed at age ≤60. The family-based *PRS* arm comprised men from families with at least two PCa cases, one of whom was diagnosed at age ≤65, or three family members diagnosed at any age. Probands provided a saliva or blood sample for genotyping and information on cancer family history through a questionnaire. Clinical data were provided by the participants’ healthcare providers.

We included families of probands genotyped for the *HOXB13* G84E mutation. To ensure consistency with sequential ascertainment rules [31] to obtain unbiased risk estimates, the analysis was based on systematically collected data from the proband, first-degree relatives (FDRs), and second-degree relatives (SDRs).

A previous case-control study has reported on PCa risks for G84E carriers using the UKGPCS case probands compared with healthy controls [8]. In the present kin-cohort study, we used data on the relatives of the probands and complex segregation analysis, and therefore this represents an independent dataset and analysis.

All participants provided written informed consent. The study was approved by the local medical research and ethics committees.

#### 2.2.2 Genotyping

Genotyping was conducted using the Infinium OncoArray-500 K BeadChip (Illumina, San Diego, CA, USA), comprising single nucleotide polymorphisms (SNPs) for genome-wide coverage and custom SNP content selected across multiple consortia based on suspected associations with one of five common cancers [32]. The *HOXB13* G84E SNP call rate was 99.99% [33]. Mutation frequencies in the probands were consistent with Hardy-Weinberg equilibrium proportions (exact test, *p* = 0.2).

### 2.3 Statistical analysis

We followed male family members from age 35 until age at PCa diagnosis, diagnosis of other cancers (excluding nonmelanoma skin cancer), death, or age 85, whichever occurred first. To handle incomplete information, we imputed missing dates of birth from FDRs’ years of birth, assuming 30 yr between subsequent generations. When age at diagnosis was missing, we assumed that PCa occurred at age of death if available, or otherwise at an age sampled from the observed age-at-diagnosis distribution of men born in the same decade from the PRM arm, or at their age at the proband's study entry, whichever was lower. We imputed missing mortality information by sampling from historical distributions of remaining lifespan past age 20 (males, England and Wales, 1841–2013; Human Mortality Database, http://www.mortality.org/).

We fitted genetic models wherein we assumed the effect of *HOXB13* G84E to follow various modes of Mendelian inheritance. We assumed single gene models where *HOXB13* G84E was the only genetic determinant and models that also included a polygenic component to allow for the fact that *HOXB13* G84E cannot explain all the familial aggregation of PCa. A polygenic component is a residual term that approximates the combined effect of a large number of unobserved low-risk alleles, in order to capture the residual familial aggregation of PCa not explained by *HOXB13* G84E. The polygenotype was assumed to be normally distributed with mean zero and was approximated using the hypergeometric polygenic model with three alleles [34], [35].

Models were parameterised in terms of the log-transformed average RRs over the polygenotype, the logit-transformed population risk allele frequency, and the log-transformed polygenic component's standard deviation (SD). We constrained the average PCa incidence across all genotypes to agree with calendar-period- and birth-cohort–specific PCa incidences for England and Wales [36] (Cancer incidence in five continents, volumes I–X, International Agency for Research on Cancer, http://ci5.iarc.fr/; Office for National Statistics, https://www.ons.gov.uk/). Details of the model parameterisation are given in the Supplementary material (model parameterisation). We assessed effect modification by allowing RRs to vary by age and birth cohort. To assess the impact of residual family history on mutation carriers specifically, we fitted models where the polygenic component was assumed not to act on mutation carriers or models that allowed separate polygenic SDs for carriers and noncarriers.

To adjust for ascertainment, we used the ascertainment-assumption-free approach [37], by modelling the conditional likelihood of observing the family phenotypes and *HOXB13* G84E mutation status, given the data relevant to ascertainment: PCa phenotype of the proband for the PRM and PRY families, and all family members’ PCa phenotypes for the PRS families.

Model comparisons were based on the Akaike information criterion (AIC) or likelihood ratio tests between nested models where appropriate. Based on the best-fitting model, we estimated absolute PCa risks based on representative examples of family history [36]. We carried out a number of sensitivity analyses, where we refitted the model to subgroups, and explored alternative imputation and censoring schemes for missing ages at diagnosis.

For model fitting, we used the pedigree analysis software MENDEL (version 3.3) [38]. All other statistical analysis was performed using R (version 3.4.0) [39].

## 3 Results

### 3.1 Systematic review and meta-analysis

**Fig. 1:**
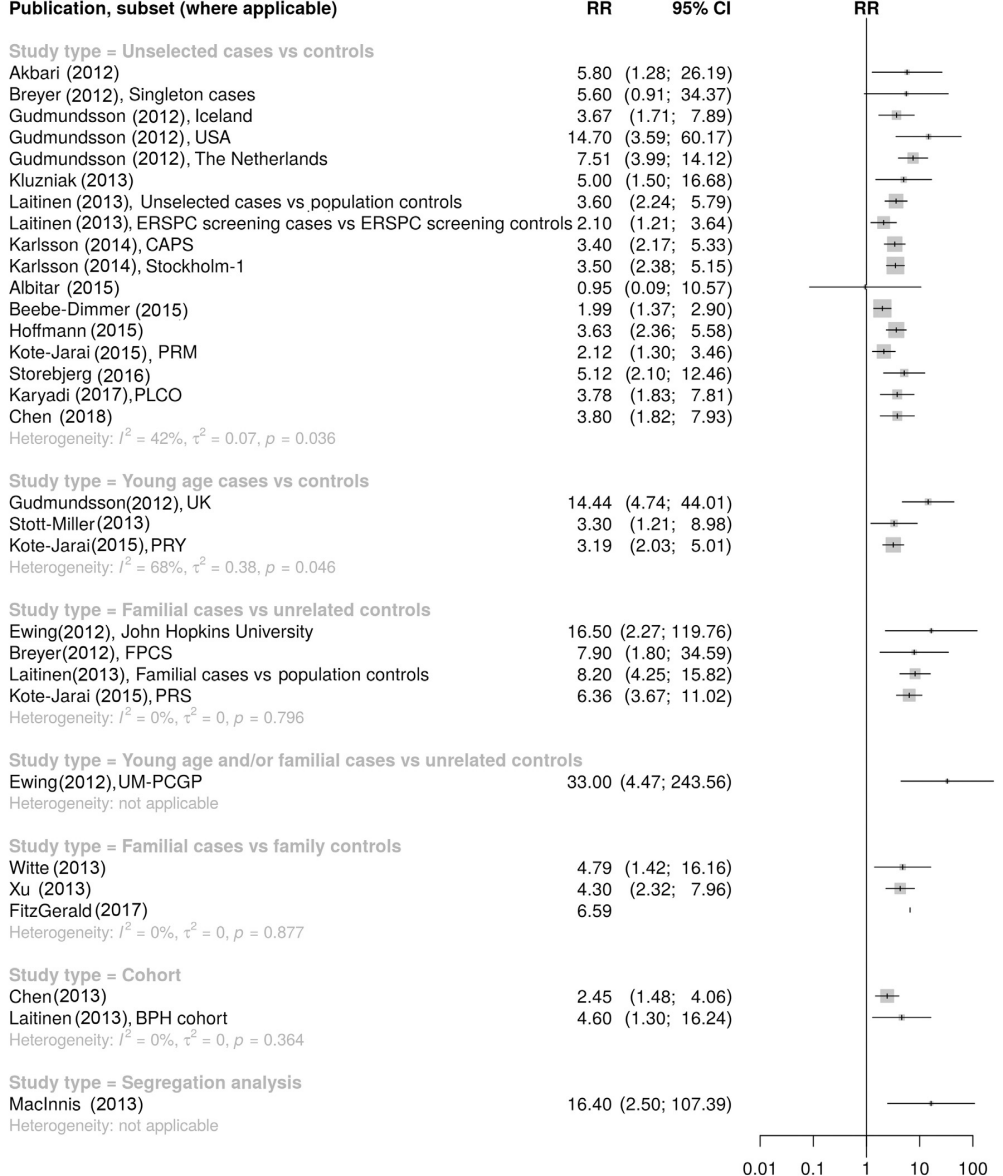
Forest plot of previous estimates of the relative risk of prostate cancer for *HOXB13* G84E mutation carriers, by study design and case selection [3], [4], [5], [6], [7], [8], [9], [10], [11], [12], [13], [14], [15], [16], [17], [18], [19], [20], [21], [22]. CI = confidence interval; RR = relative risk.

The systematic review of original research articles identified 20 publications that estimated RRs of PCa for *HOXB13* G84E carriers ([3], [4], [5], [6], [7], [8], [9], [10], [11], [12], [13], [14], [15], [16], [17], [18], [19], [20], [21], [22]; Supplementary Fig. 1). RRs from case-control studies varied between 0.95 and 33.0 (*I*^2^ = 52%, *p* < 0.001; Fig. 1 and Supplementary Table 1) but differed by case selection criteria (test for subgroup differences, *p* = 0.007). A subgroup analysis restricted to unselected case-control studies revealed significant heterogeneity between estimates (*I*^2^ = 42%, *p* = 0.036; Fig. 1) and indications of funnel plot asymmetry (*p* = 0.12; Supplementary Fig. 2). Based on unselected case-control studies, the random-effect RR estimate was 3.43 (95% CI 2.78–4.23). Omission of one outlier study yielded lower heterogeneity between the estimates (*I*^2^ = 25%, *p* = 0.2; Supplementary Table 2) but similar indications of funnel plot asymmetry (*p* = 0.11; Supplementary Fig. 3), with a random-effect RR estimate of 3.60 (95% CI 2.97–4.38; Supplementary material, systematic review and meta-analysis).

### 3.2 UKGPCS families

**Fig. 2:**
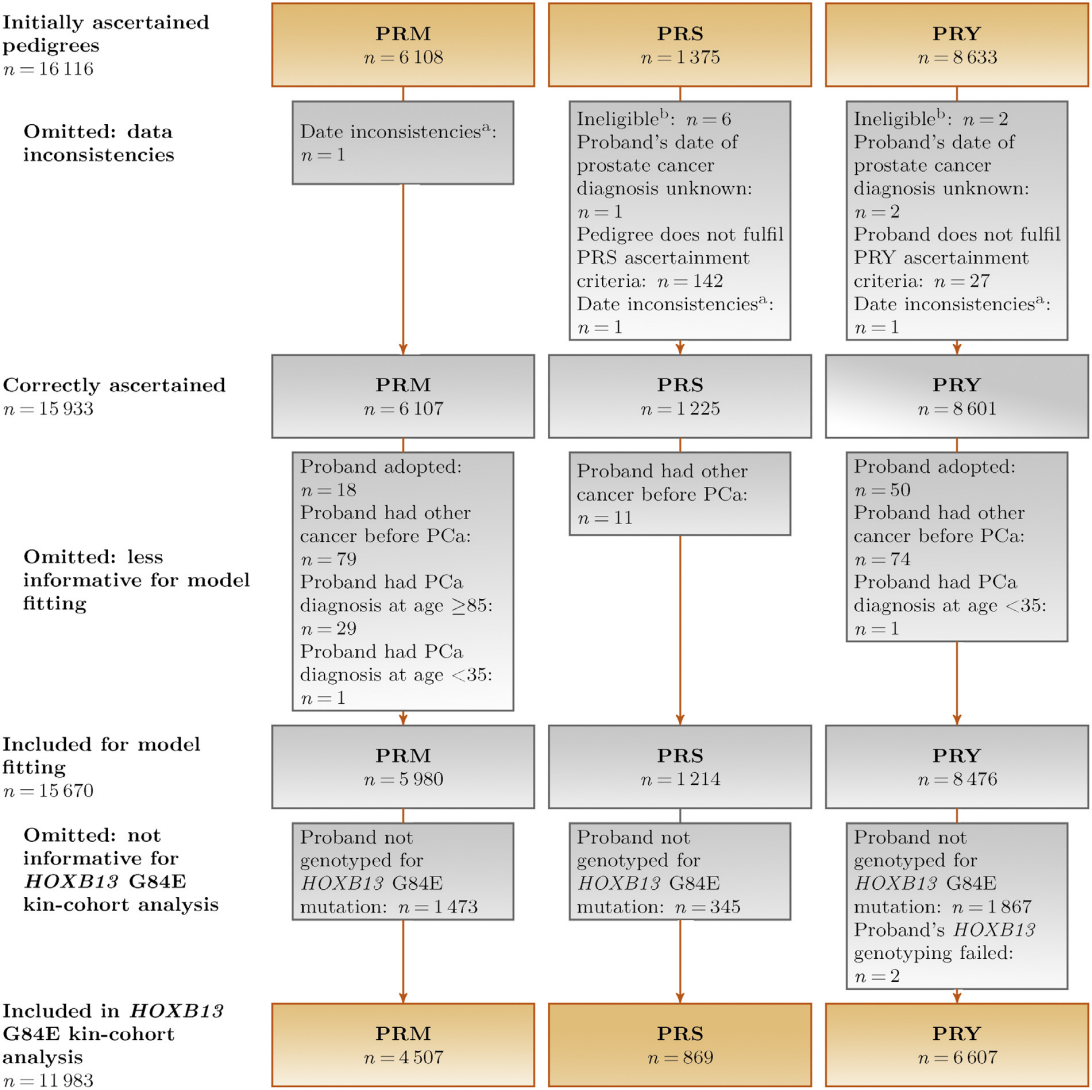
Flowchart of UKGPCS families included in the present study. *HOXB13* *=* *homeobox B13;* PCa = prostate cancer; UKGPCS = United Kingdom Genetic Prostate Cancer Study. ^a^Registered study consent date >1 yr before diagnosis. ^b^Seven without PCa, one duplicate participant.

The proband had been genotyped for *HOXB13* G84E in 11 983 of 15 670 (76%) eligible families (Fig. 2). Table 1 summarises the probands’ and family members’ characteristics. One hundred and eighty-three probands (1.5%) carried the mutation, two of whom were homozygous carriers; the proportion of mutation carriers was highest in the family-based PRS arm (2.6%) and lowest in the population-based PRM arm (1.1%). In total, 45% of mutation carriers had at least one relative who had developed PCa compared with 30% of noncarriers; these differences were most apparent in the young-onset PRY arm. Most participants were of European ancestry regardless of carrier status. Age at diagnosis was available for 62% of FDRs and 31% of SDRs with PCa; we imputed missing ages.

**Table 1:** Characteristics of included probands and families

	All		Ascertainment group					
			PRM^a^		PRS^b^		PRY^c^	
Total no. of families	11 983		4507		869		6607	
Probands’ *HOXB13* G84E status	Noncarrier	Carrier	Noncarrier	Carrier	Noncarrier	Carrier	Noncarrier	Carrier
	11 800	183	4457	50	846	23	6497	110
**Probands’ characteristics**								
Genetic ethnic ancestry								
European	10 978 (93%)	174 (95%)	4087 (92%)	47 (94%)	715 (85%)	21 (91%)	6176 (95%)	106 (96%)
African	295 (2.5%)	0 (0%)	194 (4.4%)	0 (0%)	4 (0.5%)	0 (0%)	97 (1.5%)	0 (0%)
Asian	118 (1.0%)	1 (0.5%)	94 (2.1%)	1 (2.0%)	3 (0.4%)	0 (0%)	21 (0.3%)	0 (0%)
Mixed	49 (0.4%)	0 (0%)	31 (0.7%)	0 (0%)	2 (0.2%)	0 (0%)	16 (0.2%)	0 (0%)
Unknown	360 (3.1%)	8 (4.4%)	51 (1.1%)	2 (4.0%)	122 (14%)	2 (8.7%)	187 (2.9%)	4 (3.6%)
Age at prostate cancer diagnosis (yr)								
≤44	104 (0.9%)	3 (1.6%)	11 (0.2%)	0 (0%)	1 (0.1%)	0 (0%)	92 (1.4%)	3 (2.7%)
45–54	2232 (19%)	43 (23%)	263 (5.9%)	2 (4.0%)	10 (1.2%)	0 (0%)	1959 (30%)	41 (37%)
55–64	6162 (52%)	91 (50%)	1317 (30%)	15 (30%)	399 (47%)	10 (43%)	4446 (68%)	66 (60%)
65–74	2612 (22%)	37 (20%)	2219 (50%)	29 (58%)	393 (46%)	8 (35%)	0 (0%)	0 (0%)
75–84	684 (5.8%)	8 (4.4%)	647 (15%)	4 (8.0%)	37 (4.4%)	4 (17%)	0 (0%)	0 (0%)
≥85^d^	6 (0.1%)	1 (0.5%)	0 (0%)	0 (0%)	6 (0.7%)	1 (4.3%)	0 (0%)	0 (0%)
Median (interquartile range)	59 (56–66)	58 (54.5–64.5)	67 (62–72)	67.5 (62–70)	65 (62–68)	65 (62.5–71.5)	57 (54–58)	56 (52–58)
Mean (standard deviation)	60.5 (7.7)	59.5 (8.0)	66.8 (7.3)	66.2 (6.6)	65.4 (5.1)	67.4 (7.3)	55.6 (3.8)	54.8 (4.4)
Year of prostate cancer diagnosis								
≤1994	614 (5.2%)	9 (4.9%)	463 (10%)	7 (14%)	52 (6.1%)	0 (0%)	99 (1.5%)	2 (1.8%)
1995–1999	1158 (9.8%)	20 (11%)	703 (16%)	9 (18%)	113 (13%)	3 (13%)	342 (5.3%)	8 (7.3%)
2000–2004	3009 (26%)	40 (22%)	1304 (29%)	12 (24%)	214 (25%)	6 (26%)	1491 (23%)	22 (20%)
2005–2009	3728 (32%)	60 (33%)	1321 (30%)	15 (30%)	236 (28%)	10 (43%)	2171 (33%)	35 (32%)
2010–2014	3291 (28%)	54 (30%)	666 (15%)	7 (14%)	231 (27%)	4 (17%)	2394 (37%)	43 (39%)
**Family characteristics**								
No. of 1st- and 2nd-degree relatives with prostate cancer below age 85 per family								
0	8267 (70%)	101 (55%)	3765 (84%)	41 (82%)	3 (0.4%)^e^	0 (0%)	4499 (69%)	60 (55%)
1	2435 (21%)	51 (28%)	553 (12%)	6 (12%)	418 (49%)	11 (48%)	1464 (23%)	34 (31%)
2	807 (6.8%)	26 (14%)	106 (2.4%)	3 (6.0%)	303 (36%)	9 (39%)	398 (6.1%)	14 (13%)
≥3	291 (2.5%)	5 (2.7%)	33 (0.7%)	0 (0%)	122 (14%)	3 (13%)	136 (2.1%)	2 (1.8%)
**Male 1st- and 2nd-degree relatives’ characteristics**								
Total no. with prostate cancer below age 85/total no. (%)	4572/78 583 (5.8%)	115/1315 (8.7%)	765/21 041 (3.6%)	11/240 (4.6%)	1321/8020 (16%)	40/235 (17%)	2486/49 522 (5.0%)	64/840 (7.6%)
Age at prostate cancer diagnosis								
≤44	13	1	2	0	5	0	6	1
45–54	188	4	17	1	51	0	120	3
55–64	766	22	90	0	316	13	360	9
65–74	940	28	130	4	320	7	490	17
75–84	515	11	77	1	133	6	305	4
≥85^f^	104	1	31	0	26	0	47	1
Unknown	2150	49	449	5	496	14	1205	30
Median (interquartile range)	70 (63–78)	70 (62–76)	71 (65–79)	69.5 (63–76.5)	68 (62–76)	65 (62–77)	71 (63–78)	70 (63–76)
Mean (standard deviation)	71.2 (10.7)	68.5 (10.6)	71.9 (10.6)	69.0 (12.7)	68.9 (10.1)	68.3 (9.3)	70.3 (10.9)	68.5 (11.0)
Year of birth^g^								
≤1909	809/22 644 (3.6%)	17/363 (4.7%)	231/8074 (2.9%)	1/87 (1.1%)	247/2662 (9.3%)	9/86 (10%)	331/11 908 (2.8%)	7/190 (3.7%)
1910–1919	815/10 392 (7.8%)	23/169 (14%)	146/2443 (6.0%)	2/40 (5.0%)	219/1079 (20%)	3/31 (9.7%)	450/6870 (6.6%)	18/98 (18%)
1920–1929	1142/9516 (12%)	25/177 (14%)	164/1592 (10%)	2/17 (12%)	203/573 (35%)	7/14 (50%)	775/7351 (11%)	16/146 (11%)
1930–1939	873/5040 (17%)	21/83 (25%)	129/1432 (9.0%)	3/15 (20%)	324/561 (58%)	7/10 (70%)	420/3047 (14%)	11/58 (19%)
1940–1949	660/4421 (15%)	19/78 (24%)	74/1189 (6.2%)	2/14 (14%)	266/551 (48%)	13/21 (62%)	320/2681 (12%)	4/43 (9.3%)
1950–1959	237/4519 (5.2%)	7/77 (9.1%)	17/1136 (1.5%)	1/14 (7.1%)	56/466 (12%)	1/11 (9.1%)	164/2917 (5.6%)	5/52 (9.6%)
≥1960	36/21 949 (0.2%)	3/365 (0.8%)	4/5120 (0.1%)	0/53 (0%)	6/2123 (0.3%)	0/61 (0%)	26/14 706 (0.2%)	3/251 (1.2%)
*HOXB13* G84E status^h^								
Noncarrier	431/469 (92%)	5/6 (83%)	47/56 (84%)	0/1 (0%)	183/194 (94%)	1/1 (100%)	201/219 (92%)	4/4 (100%)
Carrier	5/5^i^ (100%)	8/9 (89%)	1/1 (100%)	2/2 (100%)	2/2^i^ (100%)	4/5 (80%)	2/2 (100%)	2/2 (100%)
Not genotyped	4136/78 104 (5.3%)	102/1301 (7.8%)	717/20 980 (3.4%)	9/238 (3.8%)	1136/7826 (15%)	35/229 (15%)	2283/49 298 (4.6%)	58/834 (7.0%)

*HOXB13* *=* *homeobox B13*.

a Population-based arm: men diagnosed or treated at the Royal Marsden NHS Foundation Trust at any age.

b Family-based arm: men from families with at least two prostate cancer cases, one of whom were diagnosed at age 65 or earlier, or three family members diagnosed at any age.

c Young-onset arm: men diagnosed at 60 or earlier.

d Families in the family-ascertained PRS arm in which the proband had prostate cancer above the censoring endpoint of age 85 were retained in the study, in line with the cohort's ascertainment criteria that allow entry of families where at least three members were diagnosed at any age.

e Fulfilled inclusion criteria for the PRS arm by having third-degree or more distant relatives with prostate cancer.

f Men with prostate cancer at or above age 85 were censored at age 85. Therefore, they are not included in the total number of men with prostate cancer elsewhere in this table.

g The table shows years of birth after imputation of missing values using the years of birth of each individual's first-degree relatives and assuming a gap of 30 yr between subsequent generations.

h Genotyping data were available for 489 male relatives, of whom 14 were heterozygous mutation carriers.

i Includes two mutation carriers from the same family.

### 3.3 Model fitting and PCa risks

Table 2 summarises the results of the model-fitting process, and the Supplementary material (all models) describes the detailed results for all considered models. Under both single-gene and polygenic models, the observed family data were most consistent with a multiplicative effect of each mutation copy on PCa risk (single-gene multiplicative model, AIC = 44 312.8; polygenic multiplicative model, AIC = 40 616.1; Table 2). However, multiplicative, dominant and general models of inheritance provided similar fit (Supplementary Table 3), most likely due to the low number of homozygous carriers. When a familial polygenic component was included to allow for the residual familial effects not explained by *HOXB13* G84E mutations, the model fit improved (Table 2). This model included calendar-period- and cohort-specific incidences that capture the changing PCa incidence over time. A model in which incidences from a single calendar period (2015) were instead assumed to apply to all family members had a worse fit (AIC = 83 526.5; Table 2; Supplementary Table 4).

**Table 2:** Models for the prostate cancer incidence fitted using complex segregation analysis

Main effects models by assumed inheritance											
Model	Log likelihood	No. of parameters	AIC	Likelihood ratio test *p* value	*HOXB13* G84E	RR	95% CI	Minor allele frequency (%)	95% CI	Polygenic standard deviation	95% CI
Sporadic	−22 182.9	1	44 367.8	–		1.00^a^		0.77	0.67–0.89	0.00^a^	
Multiplicative	−22 154.4	2	44 312.8	<0.001^b^	Per allele	3.79	3.00–4.80	0.18	0.13–0.24	0.00^a^	
Polygenic	−20 315.6	2	40 635.2	<0.001^b^		1.00^a^		0.77	0.67–0.89	2.73	2.65–2.81
Polygenic multiplicative	−20 305.0	3	40 616.1	<0.001^c^	Per allele	3.86	2.16–6.88	0.20	0.11–0.36	2.72	2.64–2.80

Polygenic multiplicative model, alternative fits											
Model	Log likelihood	No. of parameters	AIC	Likelihood ratio test *p* value	Group	Per-allele RR	95% CI	Minor allele frequency (%)	95% CI	Polygenic standard deviation	95% CI
Assuming latest available population incidence (2015) for all birth cohorts	−41 760.2	3	83 526.5	–		4.20	3.49–5.04	0.14	0.10–0.18	3.26	3.23–3.30
Assuming that polygenic component does not act on mutation carriers	−20 330.2	3	40 666.4	–		9.73	7.74–12.2	0.09	0.07–0.11		
					Noncarriers					2.72	2.64–2.80
					G84E carriers					0.00^a^	
Assuming separate polygenic standard deviation for mutation carriers	−20 304.4	4	40 616.8	0.3^d^; <0.001^e^		4.98	2.74–9.05	0.16	0.09–0.29		
					Noncarriers					2.72	2.64–2.80
					G84E carriers					3.28	2.63–4.09

Polygenic multiplicative models, RR modified by age and/or birth cohort											
Model	Log likelihood	No. of parameters	AIC	Likelihood ratio test *p* value	Group	Per-allele RR	95% CI	Minor allele frequency (%)	95% CI	Polygenic standard deviation	95% CI
Age-specific RR: per year of age (log-linear model)^f^^,^^g^	−20 303.4	4	40 614.8	0.068^d^				0.18	0.11–0.31	2.72	2.64–2.80
					Baseline (age 70)	3.70	2.23–6.14				
					Per year of age	0.98	0.97–1.00				
Birth-cohort–specific RR: dichotomous birth cohort groups^h^	−20 300.8	4	40 609.6	0.004^d^				0.14	0.09–0.21	2.72	2.65–2.80
					Born ≤1929	3.09	2.03–4.71				
					Born ≥1930	5.96	4.01–8.88				
Age- and birth-cohort–specific RR^i^	−20 300.7	5	40 611.3	0.020^j^; 0.6^k^				0.14	0.09–0.21	2.72	2.65–2.80
					Born ≤1929 (age 70)	3.13	1.96–5.01				
					Born ≥1930 (age 70)	5.71	3.71–8.78				
					Per year of age	1.00	0.98–1.01				

AIC = Akaike information criterion; CI = confidence interval; *HOXB13* *=* *homeobox B13*; RR = relative risk.

a Constrained to constant value.

b Compared with sporadic model.

c Compared with polygenic model.

d Compared with polygenic multiplicative model.

e Compared with polygenic multiplicative model where the polygenic component does not act on mutation carriers.

f The model was specified as $\text{ln}\;\bar{\text{RR}}(t)=\alpha_{0}+\alpha_{1}\times (t-70)$ for men at age t, where *α*_0_ corresponds to the estimated RR at age 70 and *α*_1_ the change in RR per year of age; see the Supplementary material (model parameterisation).

g Best fitting model with age-specific RR as selected by AIC; all considered models are given in the Supplementary Table 5.

h Best fitting model with birth-cohort–specific RR as selected by AIC; all considered models are given in the Supplementary Table 5.

i The model was specified as $\text{ln}\;\bar{\text{RR}}(t,k)=\gamma_{\leq 1929}\times 1_{\left\{k\in \text{birth cohort group}\leq 1929\right\}}+\gamma_{\geq 1930}\times 1_{\left\{k\in \text{birth cohort group}\geq 1930\right\}}+\alpha \times \left(t-70\right)$ for men in birth cohort k at age t, where γ_≤_ _1929_ and γ_≥_ _1930_ correspond to the estimated RR at age 70 for men born in 1929 or earlier and in 1930 or later, respectively, and α is the change in RR per year of age; see the Supplementary material (model parameterisation).

j Compared with polygenic multiplicative model with log-linear age-specific RR.

k Compared with polygenic multiplicative model RR specific to birth cohorts ≤1929/≥1930.

Thus, we chose the multiplicative polygenic model, with calendar-period- and cohort-specific incidences as the main model for all subsequent analyses. Under this model, the average per-allele RR was 3.86 (95% CI 2.16–6.88), with a risk allele frequency of 0.20% (95% CI 0.11–0.36%) and a polygenic SD of 2.72 (95% CI 2.64–2.80). In this model, the polygenic component is assumed to act multiplicatively with *HOXB13* G84E. We tested this assumption by fitting a model that did not allow for this multiplicative effect (ie, by assuming a polygenic SD of 0 for mutation carriers), which had a significantly worse fit (*p* < 0.001). Fitting a model with separate polygenic SDs, one for mutation carriers and one for noncarriers, provided no significant evidence that the magnitude of the polygenic SD differs between mutation carriers and noncarriers (*p* = 0.3; Table 2; Supplementary Table 4).

We fitted models where the RR for mutation carriers was allowed to vary with age. None of these improved the model fit significantly, but point estimates indicated higher RRs at younger ages (Supplementary Table 5). The best-fitting model with age-specific RRs was a model that allowed the RR to vary continuously with age (*p* = 0.068), with estimated RRs of 5.07 at age 50 that decreased to 3.70 at age 70 (Table 2). A model with separate RRs for mutation carriers in the seven assumed birth cohorts showed higher RR estimates for men born more recently and fitted significantly better than the main polygenic multiplicative model where a single RR was assumed to apply to all mutation carriers (*p* = 0.014; Supplementary Table 5). However, the best fitting model with cohort-specific RRs, as determined by AIC, was a model that included an RR parameter for men born in 1929 or earlier and a separate RR parameter for men born in 1930 or later (AIC = 40609.6, *p* = 0.004 compared with the main polygenic multiplicative model; Table 2; Supplementary Table 5). Finally, a model that allowed for both age- and cohort-specific RRs did not have improved fit compared with the model with cohort-specific RRs (*p* = 0.7; Table 2). Thus, the model with birth-cohort–specific RRs was the most parsimonious; in this model, the estimated per-allele RR was 3.09 (95% CI 2.03–4.71) for men born in 1929 or earlier and 5.96 (95% CI 4.01–8.88) for men born in 1930 or later, with a risk allele frequency of 0.14% (95% CI 0.09–0.21%) and a polygenic SD of 2.72 (95% CI 2.65–2.80).

**Fig. 3:**
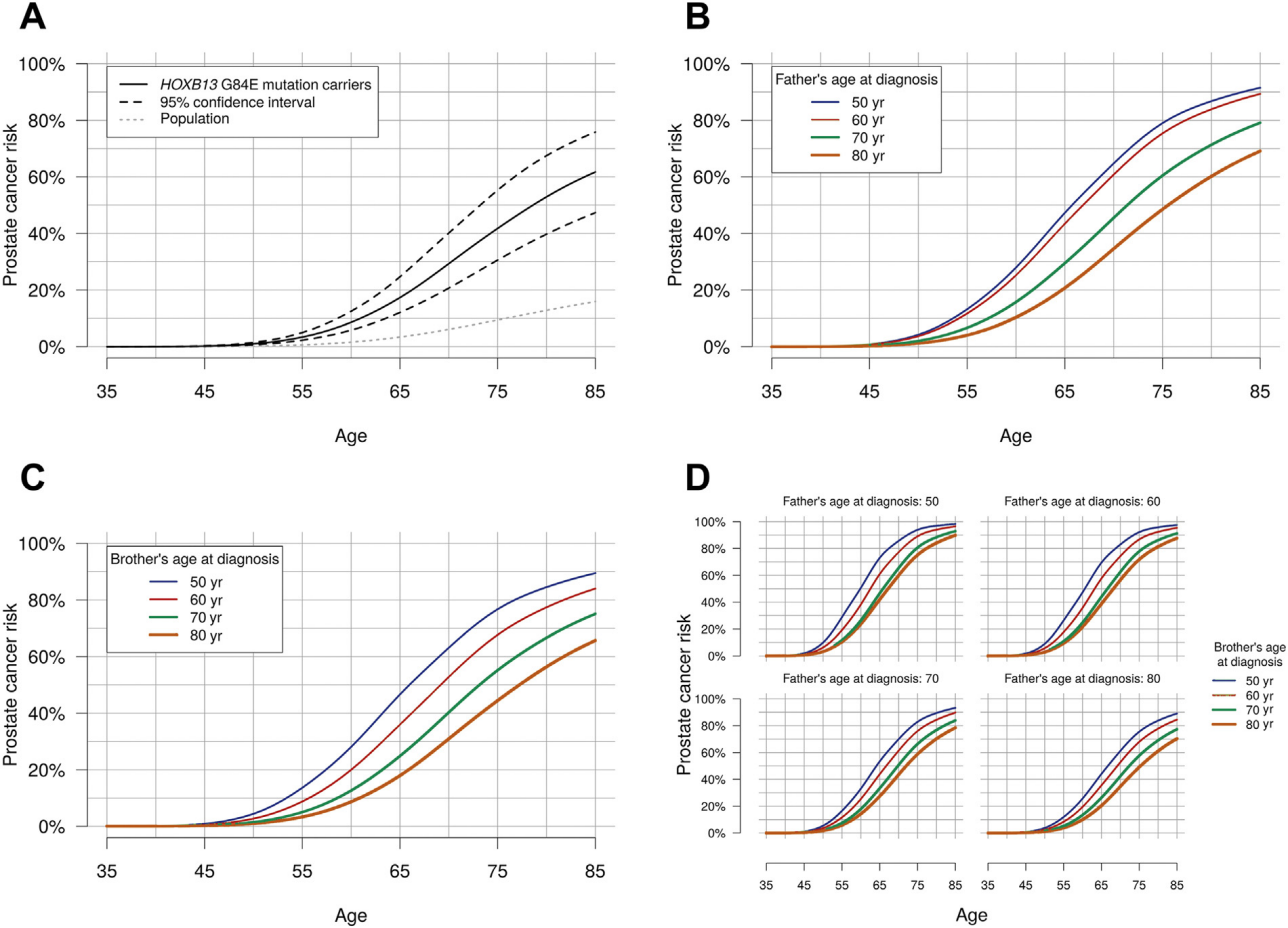
Predicted cumulative prostate cancer risks for a 35-yr-old man born in 1960 or later and carrying a single copy of the *HOXB13* G84E mutation, as estimated by the most parsimonious model, with (A) average (unknown) prostate cancer family history, with a 95% confidence interval; (B) a father diagnosed with prostate cancer at varying ages; (C) a brother diagnosed with prostate cancer at varying ages; and (D) a father and a brother diagnosed with prostate cancer at varying ages. In all family history scenarios, consultands and their brothers were assumed to be born in 1960 or later, and fathers were assumed to be born in 1930–39. *HOXB13* *=* *homeobox B13.*

Table 3 and Fig. 3 show the predicted age-specific risks of developing PCa for a *HOXB13* G84E mutation carrier born in 1960 or later, based on the most parsimonious model and under different assumptions about PCa family history. The average predicted PCa risk by age 85 is 62% (95% CI 47–76%) for *HOXB13* G84E mutation carriers, compared with 15% for noncarriers. For a mutation carrier with an affected father, the corresponding risk estimate ranges from 69% to 92% depending on the father's age at diagnosis, and for a man with two affected FDRs, the risk estimate ranges from 70% to 98%. The predicted average risks for mutation carriers born prior to 1960 are shown in Supplementary Figure 4.

**Table 3:** Predicted cumulative prostate cancer risks for a 35-yr-old man born in 1960 or later and carrying a single copy of the *HOXB13* G84E mutation, as estimated by the most parsimonious model, by varying family history of prostate cancer

Family history	Prostate cancer risk for consultand by age (yr)									
	40	45	50	55	60	65	70	75	80	85
Average familial risk^a^	0.0%	0.2%	1%	3%	9%	17%	29%	42%	53%	62%
Father unaffected at age 80, grandfather unaffected at age 80	0.0%	0.1%	0.6%	2%	6%	14%	25%	38%	50%	60%
Father unaffected at age 80, grandfather had prostate cancer at age 80	0.0%	0.2%	0.9%	3%	9%	19%	33%	48%	60%	70%
Father had prostate cancer at age 80, grandfather had prostate cancer at age 80	0.0%	0.3%	1%	5%	12%	25%	40%	56%	69%	78%
Father had prostate cancer at age (yr)										
40	0.1%	0.8%	4%	13%	28%	47%	65%	79%	87%	92%
50	0.1%	0.8%	4%	13%	28%	47%	65%	79%	87%	92%
60	0.1%	0.7%	4%	12%	25%	43%	61%	75%	84%	89%
70	0.0%	0.4%	2%	7%	16%	29%	46%	60%	71%	79%
80	0.0%	0.2%	1%	4%	10%	21%	35%	49%	60%	69%
Brother had prostate cancer at age (yr)										
40	0.1%	0.9%	5%	14%	29%	48%	65%	78%	86%	90%
50	0.1%	0.9%	4%	14%	28%	47%	63%	77%	85%	89%
60	0.1%	0.5%	3%	9%	20%	36%	53%	68%	77%	84%
70	0.0%	0.3%	1%	5%	13%	25%	40%	55%	67%	75%
80	0.0%	0.2%	0.9%	3%	9%	18%	31%	44%	56%	66%

Prostate cancer risk for consultand by age 85 yr					
	Brother had prostate cancer at age (yr)				
	40	50	60	70	80
Father had prostate cancer at age (yr)					
40	98%	98%	97%	93%	90%
50	98%	98%	96%	93%	90%
60	98%	98%	95%	91%	88%
70	94%	93%	90%	84%	78%
80	90%	89%	84%	77%	70%

*HOXB13* = *homeobox B13*.

In all family history scenarios, consultands and their brothers were assumed to be born in 1960 or later, fathers were assumed to be born in 1930–39, and grandfathers were assumed to be born in 1909 or earlier.

a That is, ignoring family history information.

The Supplementary material (sensitivity analyses) shows the results of our sensitivity analyses. Restriction to FDRs produced similar results as for the main analysis (Supplementary Table 6), as did alternative age imputation schemes (Supplementary Table 7). Censoring all family members with missing ages at diagnosis at age 0 resulted in lower point estimates but similar results with respect to the higher RR estimates for men born in 1930 and later (Supplementary Table 7). Splitting the data by the study arm yielded somewhat higher RR estimates when the model was fitted using only the PRY compared with the PRM families (Supplementary Table 6).

## 4 Discussion

The use of genetic information is becoming increasingly important in urological practice, both to estimate risk and to target treatments. It is therefore crucial to have precise risk estimates for the known PCa susceptibility variants, including mutations in *HOXB13*. We have performed a systematic review of published risk estimates for *HOXB13* G84E mutation carriers, and using the largest PCa case-family dataset available to date, we have estimated age-specific PCa risks for mutation carriers and investigated variation in these risks by PCa family history. The pooled RR estimate from a meta-analysis restricted to unselected case-control studies was 3.43 (95% CI 2.78–4.23), consistent with the RR estimate from the present study of 3.86 (95% CI 2.16–6.88). Based on our data, the average absolute PCa risk for mutation carriers is 62% (95% CI 47–76%) by age 85 for men born in 1960 or later. Models that allowed for a polygenic-modifying component on these risks fitted significantly better, suggesting that other familial factors, that is, genetic, environmental, and/or lifestyle-related effect modifiers that cluster in families, modify the PCa risk for mutation carriers. The results suggest that the PCa family history should be taken into account in the genetic counselling process of *HOXB13* G84E mutation carriers and that a single set of penetrance estimates would not be applicable to all mutation carriers. We have presented absolute risk estimates both applicable to the average mutation carrier in the UK population and tailored to men with family history configurations typically seen in family clinics.

We used calendar-period- and birth-cohort–specific population PCa incidences that account for the rising population incidences over time. This may be particularly important given the recent more widespread use of prostate-specific antigen (PSA) testing. Despite this, RR estimates for mutation carriers were higher in more recent birth cohorts, over and above the general rise in population incidences. For example, the predicted PCa risks by age 85 are 19%, 54%, and 62% for mutation carriers born in 1909 or earlier, during 1930–1939, and in 1960 or later, respectively. This could reflect a true birth cohort effect, resulting from changes in environmental or lifestyle factors over time. Alternatively, it may also result from the possibility that men with affected relatives may be more likely to request a PSA test and that the increased availability of PSA testing thus might have resulted in clustering of PCa diagnoses in more recent generations. One way to evaluate this potential source of bias would be to assess the association with the risk of aggressive or fatal PCa only. Unfortunately, PCa in relatives was reported by the probands and data on tumour aggressiveness in relatives were not available to us. We note, however, that previous studies revealed at most minor differences in tumour aggressiveness between carriers and noncarriers of *HOXB13* G84E mutation [5], [6], [7], [8], [9], [10], [11], [12], [14], [16], [20], [21] and that familial RRs of PCa estimated on the basis of the UKGPCS population-based arm were in line with the estimates from other large epidemiological studies [40].

Previous studies reported higher RRs at younger ages [3], [5], [6], [10]. We found some evidence of decreasing RRs with age, but a model with both age- and cohort-specific RRs did not fit significantly better than a model with only cohort-specific RRs and estimated an adjusted per-year-of-age RR of 1.00 (95% CI 0.98–1.01). This could however be due to lack of power, and our results suggest that it is difficult to distinguish between decreasing age-specific RRs and increasing risks with more recent birth cohort because the effects are confounded. Only one other study investigated variation in RRs by birth cohort but did not observe significant differences [13]; however, it was based on only 19 families with mutations.

The meta-analysis of previous studies revealed significant differences in reported RRs by case selection, in particular, when case selection depended on family history. This is consistent with the results of the present analysis where we found that PCa risks for mutation carriers vary by PCa family history and suggest that model-based estimates, which consider family history similar to those presented here, may be more appropriate for counselling purposes. Furthermore, among unselected case-control studies, we found heterogeneity between published RR estimates as well as indications of funnel plot asymmetry. This may reflect differences in study design, selection criteria, adjustment variables used in the analysis, and/or publication bias [41], and caution is required in the use of the resulting pooled estimate.

Based on the most parsimonious model, approximately one in 360 individuals carries the *HOXB13* G84E mutation in the UK. This is consistent with previous UK and Western European population estimates [4], [8], [9]. Based on this mutation frequency estimate and the RR estimate for men born in 1930 or later and assuming a familial RR in FDRs of 2.5 [42], *HOXB13* G84E accounts for approximately 3.6% of the excess familial risk of PCa [43].

Family-based studies can produce high precision risk estimates, due to the aggregation of likely mutation carriers. However, because individuals are ascertained through affected family members and thus generally are at a higher than average risk of disease, adjustment for the ascertainment is needed to avoid biased estimates [24]. Among the previous family-based studies on *HOXB13* G84E and PCa risk [3], [10], [13], [19], only two adjusted for the ascertainment procedure or the relatedness between subjects [13], [19]. Here, we adjusted for ascertainment using the ascertainment-assumption-free approach, which gives unbiased estimates provided that all information related to the ascertainment is available, however at the cost of somewhat reduced precision [37].

Strengths of our study include the large sample size and the use of the kin-cohort study design, allowing the use of cancer history information in relatives of mutation carriers. The dataset included both families ascertained through population-based PCa cases and additional families enriched for a young age at diagnosis or a family history of PCa. This provided information on mutation carriers with a wide range of ages at diagnosis and family history configurations, which enabled us to model the variation in risks by family history and other characteristics. Our model-based estimates would thus be applicable to not only mutation carriers identified in family clinics, but also carriers identified through population-based mutation screening programmes.

Limitations include the reliance on self-reported cancer family history, which can be inaccurate, particularly for more distant relatives [44]. We evaluated the impact of including information on SDRs on the results by refitting the model using only FDRs, and the estimates remained similar. Furthermore, since men were unaware of their mutation status at study entry, no differential reporting of family history by mutation status should be expected. We imputed missing ages at diagnosis in relatives using the age distribution in the population-based families, which may approximate the age-at-diagnosis variation of the general population. While imputation adds uncertainty, alternative imputation schemes based on external population incidence information or those that instead allowed study-arm–specific imputations produced similar results. When all relatives with unknown ages were censored, the estimated RRs and polygenic SD were lower and likely underestimated. However, the differences in RR estimate sizes between the birth cohorts remained, indicating that these findings are not driven by the assumed imputation scheme. In subgroup analyses, the families ascertained through a young case generally showed higher RR estimates compared with the population-based families, which may reflect a residual bias even after the ascertainment adjustment. Confidence intervals were overlapping and apparent subgroup differences could be due to chance, but we cannot exclude the possibility that imperfect ascertainment adjustment may have resulted in somewhat overestimated RRs. Finally, although a multiplicative model showed best fit, the low number of homozygous mutation carriers complicates distinguishing multiplicative from dominant or general models of inheritance.

## 5 Conclusions

We have shown that the risk of PCa for *HOXB13* G84E mutation carriers varies by PCa family history and by birth cohort. The family-history- and birth-cohort–specific risks may be useful in the counselling of mutation carriers. The current estimates should be incorporated into comprehensive risk prediction models, which also consider other known genetic predisposition variants including low-risk common susceptibility alleles identified through genome-wide association studies, to enable tailored clinical risk prediction of this highly polygenic disease.

***Author contributions:*** Tommy Nyberg had full access to all the data in the study and takes responsibility for the integrity of the data and the accuracy of the data analysis.  

*Study concept and design:* Antoniou, Nyberg.

*Acquisition of data:* Kote-Jarai, Eeles, Govindasami, Leslie, Dadaev, Bancroft, Hussain, Keating, McMahon, Morgan, Mullen, Osborne, Rageevakumar, UKGPCS Collaborators.

*Analysis and interpretation of data:* Nyberg, Antoniou.

*Drafting of the manuscript:* Nyberg, Antoniou.

*Critical revision of the manuscript for important intellectual content:* Kote-Jarai, Eeles, Ni Raghallaigh, Brook, Govindasami, Leslie, Dadaev, Bancroft, Hussain, Keating, Lee, McMahon, Morgan, Mullen, Osborne, Rageevakumar.

*Statistical analysis*: Nyberg, Antoniou, Lee.

*Obtaining funding*: Antoniou, Kote-Jarai, Eeles.

*Administrative, technical, or material support*: Govindasami, Hussain, Keating, McMahon, Morgan, Mullen, Osborne, Rageevakumar.

*Supervision:* Antoniou, Kote-Jarai, Eeles.

*Other:* None.  

***Financial disclosures:*** Tommy Nyberg certifies that all conflicts of interest, including specific financial interests and relationships and affiliations relevant to the subject matter or materials discussed in the manuscript (eg, employment/affiliation, grants or funding, consultancies, honoraria, stock ownership or options, expert testimony, royalties, or patents filed, received, or pending), are the following: None.  

***Funding/Support and role of the sponsor:*** This risk modelling work was supported by the Cancer Research UK (grant numbers C12292/A20861, C12292/A22820). The UKGPCS was supported by Cancer Research UK (grant numbers C5047/A7357, C5047/A3354, C5047/A10692, C5047/A15007 and C5047/A17528). Funding support was provided also by the Prostate Research Campaign UK (now Prostate Cancer UK), The Institute of Cancer Research, The Everyman Campaign, The National Cancer Research Network UK, The National Cancer Research Institute (NCRI) UK.  

***Acknowledgements:*** We thank all the patients who took part in the UKGPCS study. We would like to acknowledge the NCRN nurses and consultants for their work in the study. We are grateful for support of NIHR funding to the NIHR Biomedical Research Centre at The Institute of Cancer Research and The Royal Marsden NHS Foundation Trust. We acknowledge the PRACTICAL consortium for organising genotyping using the OncoArray which provided data for this study (http://practical.icr.ac.uk/).
