# Supplementary material for: Homeobox B13 G84E Mutation and Prostate Cancer Risk
Source: Eur Urol. 2019 May;75(5):834–45. doi: 10.1016/j.eururo.2018.11.015 (PMC6470122; doi:10.1016/j.eururo.2018.11.015)
Supplement: Supplementary file 1 [file mmc1.pdf]

Supplementary material for
Homeobox B13 G84E mutation and prostate cancer risk

Contents

Appendix – Systematic review and meta-analysis

Supplementary Fig. 1 – Systematic review and meta-analysis: Flowchart detailing the identification of original research articles on the relative risk of prostate cancer for *HOXB13* G84E mutation carriers

Supplementary Table 1 – Systematic review and meta-analysis: Summary of published studies that estimate the relative risk of prostate cancer for *HOXB13* G84E mutation carriers

Supplementary Fig. 2 – Systematic review and meta-analysis: Funnel plot of published relative risk estimates from unselected case-control studies

Supplementary Table 2 – Systematic review and meta-analysis: Leave-one-out sensitivity analysis for the unselected case-control studies

Supplementary Fig. 3 – Systematic review and meta-analysis: Funnel plot of published relative risk estimates from unselected case-control studies, after omission of Beebe-Dimer *et al*. (2015)

Appendix – Model parameterisation

Appendix – All models

Supplementary Table 3 – All models: Main effects models by assumed inheritance

Supplementary Table 4 – All models: Polygenic multiplicative model, alternative fits

Supplementary Table 5 – All models: Polygenic multiplicative model, RR modified by age and/or birth cohort

Supplementary Fig. 4 – Predicted cumulative prostate cancer risks for a 35 year old man carrying a single copy of the *HOXB13* G84E mutation with average (unknown) prostate cancer family history, as estimated by the most parsimonious model, by birth cohort

Appendix – Sensitivity analyses

Supplementary Table 6 – Sensitivity analyses: Most parsimonious polygenic multiplicative model with birth-cohort-specific RRs, refitted to subgroups

Supplementary Table 7 – Sensitivity analyses: Most parsimonious polygenic multiplicative model with birth-cohort-specific RRs, alternative imputation schemes

Systematic review and meta-analysis

Methods

We carried out a systematic review and meta-analysis with the aim to synthesise the available evidence on the association between the *HOXB13* G84E mutation and prostate cancer (PCa) risk. Specifically, we aimed to derive a pooled estimate which reflects the strength of association for unselected men in the general population.

On 1st February 2018, we searched Pubmed, MEDLINE and Embase for original publications using the search query

("HOXB13" OR "Homeobox B13" OR "rs138213197" OR "G84E" OR "p.Gly84Glu" OR "c.251G>A") AND "Prostate"

with no time period or language restrictions.

From the publications, we extracted RR estimates (odds ratio, risk ratio or hazard ratio), and approximated standard errors from the associated confidence interval widths. When RR and/or CI had not been reported but sufficient information was otherwise available in the publication (e.g. absolute frequencies in case-control studies), we calculated missing measures. Whenever available, we used adjusted estimates as reported in the publications.

For articles that reported on multiple studies, we extracted results from the individual studies rather than results based on pooled datasets whenever possible. We contacted the authors of three publications to ask for clarifications.

We grouped the RR estimates according to study design, and for case-control studies according to case and control selection as well as other study covariates, and tested for differences by fitting models with covariate moderators. To assess heterogeneity between published estimates, we used the DerSimonian-Laird heterogeneity of effects chi-square test and corresponding I^2 statistic. We assessed potential publication bias by producing funnel plots and tested for funnel plot asymmetry using the rank correlation test. We derived pooled meta-analysis estimates according to both fixed effects and random effects models. Finally, we performed a leave-one-out sensitivity analysis to assess the impact of individual studies on the results, by omitting one of the included studies at a time and refitting the models.

The meta-analysis was done using R software (version 3.4.0), with the *meta* and *metafor* packages.

Results

We identified 121 original research articles, of which 20 presented an estimate of the RR of prostate cancer for *HOXB13* G84E mutation carriers compared to the general population or non-carriers based on original data (Supplementary Fig. 1; (Akbari *et al.*, 2012; Breyer *et al.*, 2012; Ewing *et al.*, 2012; Gudmundsson *et al.*, 2012; Kluźniak *et al.*, 2013; Laitinen *et al.*, 2013; MacInnis *et al.*, 2013; Stott-Miller *et al.*, 2013; Witte *et al.*, 2013; Xu *et al.*, 2013; Chen *et al.*, 2013, 2018; Karlsson *et al.*, 2014; Albitar *et al.*, 2015; Kote-Jarai *et al.*, 2015; Beebe-Dimmer *et al.*, 2015; Hoffmann *et al.*, 2015; Storebjerg *et al.*, 2016; Karyadi *et al.*, 2017; FitzGerald *et al.*, 2017)). Supplementary Table 1 provides a summary of these publications.

A total of 17 case-control studies from 13 publications reported estimates based on unselected cases (Akbari *et al.*, 2012; Breyer *et al.*, 2012; Gudmundsson *et al.*, 2012; Laitinen *et al.*, 2013; Kluźniak *et al.*, 2013; Karlsson *et al.*, 2014; Albitar *et al.*, 2015; Kote-Jarai *et al.*, 2015; Beebe-Dimmer *et al.*, 2015; Hoffmann *et al.*, 2015; Storebjerg *et al.*, 2016; Karyadi *et al.*, 2017; Chen *et al.*, 2018), eight studies from six publications reported estimates based on cases selected on the basis of a young age at PCa diagnosis and/or family history (Breyer *et al.*, 2012; Ewing *et al.*, 2012; Gudmundsson *et al.*, 2012; Laitinen *et al.*, 2013; Stott-Miller *et al.*, 2013; Kote-Jarai *et al.*, 2015), and three studies from three publications reported estimates based on case-family study designs (Witte *et al.*, 2013; Xu *et al.*, 2013; FitzGerald *et al.*, 2017). Among the unselected case-control studies, 15 reported RR estimates that were significantly different from 1. All RR estimates from case-control studies with other case selections showed statistically significant deviation from 1. In addition, two independent prospective cohort studies where initially PCa-negative were followed for PCa onset (Chen *et al.*, 2013; Laitinen *et al.*, 2013), and a kin-cohort study based on modified segregation analysis (MacInnis *et al.*, 2013) reported statistically significant RR estimates.

Meta-analysis

Case-control studies

Fig. 1 shows a forest plot of published RRs. Among case-control studies, the RR estimates showed moderate heterogeneity (I^2=52%, p<0.001), and differed by case selection (unselected, young age, familial, or both young age and familial cases; test for subgroup differences, p=0.007). In particular, estimates from case-control studies based on cases selected for family history were on average higher than estimates from studies on unselected cases (p=0.005).

We restricted the meta-analysis to case-control studies that compared unselected cases (with respect to age at diagnosis or family history) to unrelated controls. In these unselected case-control studies, RR estimates still showed moderate heterogeneity (I^2=42%, p=0.036), and the corresponding funnel plot showed a tendency for asymmetry with many smaller studies reporting estimates higher than average (Supplementary Fig. 2; test for asymmetry, p=0.12). Overall, based on all unselected case-control studies, we derived a fixed effects model estimate of RR=3.23 (95% CI 2.80-3.74), and a random effects model estimate of RR=3.43 (95% CI 2.78-4.23).

Within the 17 unselected case-control studies, we found no statistically significant differences by control selection (population-based, screening-negative, or other disease controls; test for subgroup differences, p=0.7), nor by population setting (Europe or North America; test for subgroup differences, p=0.8). Two of these studies relied on cases from PSA screening trials (Laitinen *et al.*, 2013; Karyadi *et al.*, 2017), while the remaining 15 studies relied on observational case series. Restriction to the studies with observational case series did not substantially change the estimated RRs (fixed effects model RR=3.32, 95% CI 2.85-3.88; random effects model RR=3.57, 95% CI 2.84-4.49) nor the estimated heterogeneity (I^2=44%, p=0.036). Two of the studies used age-matched controls and four adjusted for age and/or other potential confounders. Five did no age adjustment but relied on screening-negative controls of similar age to the cases, or controls that were otherwise of similar ages. In the remaining six studies no adjustments were done. There was no significant difference by age adjustment scheme (matching/covariate adjustment, no adjustment but cases and controls were of similar ages, or no adjustment but cases and controls were of different ages; test for subgroup differences, p=0.9). Regressing on the average (mean or median) age of cases in the unselected case-control studies[1] revealed indications of a decrease in RRs with higher average age of cases, but this trend was not statistically significant and would only explain a minor proportion of the heterogeneity between estimates (test for continuous moderator, p=0.12; residual I^2=39%, p=0.058).

In the leave-one-out sensitivity analysis, the fixed effects RR estimates varied between 3.09 and 3.52, and the random effects RR estimates between 3.18 and 3.60, and two studies were revealed to have outlier RRs whose omission had a substantial effect on the estimated heterogeneity (Supplementary Table 2; after omission, I^2=25%, p=0.2 (Beebe-Dimmer *et al.*, 2015); after omission, I^2=26%, p=0.2 (Gudmundsson *et al.*, 2012)). One of these two studies reported an RR estimate of 1.99 (95% CI 1.37-2.90), and relied on self-reported personal cancer history through a questionnaire to classify cases and controls (Beebe-Dimmer *et al.*, 2015). Misclassification of cases and controls is therefore a possible explanation for this outlying low RR estimate, as nondifferential misclassification may result in bias towards the null. We could not identify any likely methodological explanation for the outlying RR estimate of 7.51 (95% CI 3.99-14.11) from the other study (Gudmundsson *et al.*, 2012). After omission of the Beebe-Dimmer *et al* (2015) estimate, the estimated heterogeneity between the remaining 16 unselected case-control studies was low (I^2=25%, p=0.2), the fixed effects model RR estimate was 3.52 (95% CI 3.01-4.13), and the random effects model RR estimate was 3.60 (95% CI 2.97-4.38). The corresponding funnel plot however showed similar indications of asymmetry as before the omission, with several smaller studies reporting higher than average RRs (Supplementary Fig. 3; test for asymmetry, p=0.11).

---

[1] For the purposes of this analysis, we used the mean age of the cases when available, but otherwise the median age if only the median had been reported. One study only reported mean ages by case subgroups (Gudmundsson *et al.*, 2012); we estimated the global mean for cases using the corresponding subgroup sizes. One study only reported the cases' ages in age groups (Karyadi *et al.*, 2017); we approximated the mean age based on the frequencies and midpoint age of each age group. Three studies did not report the age of cases, and we used the reported mean age of cases and controls combined (Beebe-Dimmer *et al.*, 2015; Hoffmann *et al.*, 2015), or the corresponding mean age of case-probands from the current analysis of the UKGPCS dataset (Kote-Jarai *et al.*, 2015).

Other study designs

Two prospective cohort studies have been performed, among biopsy-confirmed initially PCa-negative men with moderately heightened PSA (Chen *et al.*, 2013) and among biopsy-confirmed initially PCa-negative men with benign prostate hyperplasia (Laitinen *et al.*, 2013). While both studies relied on inclusion criteria with strong selection, we are not aware of any evidence to imply that PSA or BPH would act as effect modifiers. Finally, one previous kin-cohort study recruited cases based on young age, but adjusted for this ascertainment and analysed PCa patterns in family members at any age (MacInnis *et al.*, 2013). When these three studies were added to the previous 17 unselected case-control studies there was no substantial change in the estimated heterogeneity between estimates (I^2=40%, p=0.032). The fixed effects model RR estimate was 3.21 (95% CI 2.80-3.69), and the random effects model estimate was 3.42 (95% CI 2.81-4.17). When one outlier unselected case-control study was omitted, the corresponding fixed effects model RR estimate was 3.46 (95% CI 2.98-4.02 and the random effects model RR estimate was 3.57 (95% CI 2.96-4.30).

References

Akbari, M. R. *et al.* (2012) 'Association between germline HOXB13 G84E mutation and risk of prostate cancer', *Journal of the National Cancer Institute*. Oxford University Press, 104(16), pp. 1260–1262. doi: 10.1093/jnci/djs288.

Albitar, F. *et al.* (2015) 'Synonymous Polymorphisms in HOXB13 as a Protective Factor for Prostate Cancer.', *Journal of Cancer*. Ivyspring International Publisher, 6(5), pp. 409–11. doi: 10.7150/jca.11413.

Beebe-Dimmer, J. L. *et al.* (2015) 'The HOXB13 G84E Mutation Is Associated with an Increased Risk for Prostate Cancer and Other Malignancies.', *Cancer Epidemiology, Biomarkers & Prevention*. AACR, 24(9), pp. 1366–72. doi: 10.1158/1055-9965.EPI-15-0247.

Breyer, J. P. *et al.* (2012) 'Confirmation of the HOXB13 G84E germline mutation in familial prostate cancer.', *Cancer Epidemiology, Biomarkers & Prevention*. AACR, 21(8), pp. 1348–53. doi: 10.1158/1055-9965.EPI-12-0495.

Chen, H. *et al.* (2018) 'Genetic factors influencing prostate cancer risk in Norwegian men.', *The Prostate*. Wiley Online Library, 78(3), pp. 186–192. doi: 10.1002/pros.23453.

Chen, Z. *et al.* (2013) 'The G84E mutation of HOXB13 is associated with increased risk for prostate cancer: Results from the REDUCE trial', *Carcinogenesis*. Oxford Univ Press, 34(6), pp. 1260–1264. doi: 10.1093/carcin/bgt055.

Ewing, C. M. *et al.* (2012) 'Germline Mutations in *HOXB13* and Prostate-Cancer Risk', *New England Journal of Medicine*. Mass Medical Soc, 366(2), pp. 141–149. doi: 10.1056/NEJMoa1110000.

FitzGerald, L. M. *et al.* (2017) 'Impact of the G84E variant on HOXB13 gene and protein expression in formalin-fixed, paraffin-embedded prostate tumours.', *Scientific Reports*. Nature Publishing Group, 7(1), p. 17778. doi: 10.1038/s41598-017-18217-w.

Gudmundsson, J. *et al.* (2012) 'A study based on whole-genome sequencing yields a rare variant at 8q24 associated with prostate cancer', *Nature Genetics*, 44(12), pp. 1326–1329. doi: 10.1038/ng.2437.

Hoffmann, T. J. *et al.* (2015) 'Imputation of the rare HOXB13 G84E mutation and cancer risk in a large population-based cohort.', *PLoS Genetics*. Public Library of Science, 11(1), p. e1004930. doi: 10.1371/journal.pgen.1004930.

Karlsson, R. *et al.* (2014) 'A population-based assessment of germline HOXB13 G84E mutation and prostate cancer risk.', *European Urology*. Elsevier, 65(1), pp. 169–76. doi: 10.1016/j.eururo.2012.07.027.

Karyadi, D. M. *et al.* (2017) 'Whole exome sequencing in 75 high-risk families with validation and replication in

independent case-control studies identifies TANGO2, OR5H14, and CHAD as new prostate cancer susceptibility genes.', *Oncotarget*. Impact Journals, LLC, 8(1), pp. 1495–1507. doi: 10.18632/oncotarget.13646.

Kluźniak, W. *et al.* (2013) 'The G84E mutation in the HOXB13 gene is associated with an increased risk of prostate cancer in Poland', *The Prostate*. Wiley Online Library, 73(5), pp. 542–548. doi: 10.1002/pros.22594.

Kote-Jarai, Z. *et al.* (2015) 'Prevalence of the HOXB13 G84E germline mutation in British men and correlation with prostate cancer risk, tumour characteristics and clinical outcomes.', *Annals of Oncology*. Eur Soc Med Oncology, 26(4), pp. 756–61. doi: 10.1093/annonc/mdv004.

Laitinen, V. H. *et al.* (2013) 'HOXB13 G84E mutation in Finland: population-based analysis of prostate, breast, and colorectal cancer risk.', *Cancer Epidemiology, Biomarkers & Prevention*. AACR, 22(3), pp. 452–60. doi: 10.1158/1055-9965.EPI-12-1000-T.

MacInnis, R. J. *et al.* (2013) 'Population-based estimate of prostate cancer risk for carriers of the HOXB13 missense mutation G84E.', *PloS One*. Public Library of Science, 8(2), p. e54727. doi: 10.1371/journal.pone.0054727.

Storebjerg, T. M. *et al.* (2016) 'Prevalence of the HOXB13 G84E mutation in Danish men undergoing radical prostatectomy and its correlations with prostate cancer risk and aggressiveness.', *BJU International*. Wiley Online Library, 118(4), pp. 646–53. doi: 10.1111/bju.13416.

Stott-Miller, M. *et al.* (2013) 'HOXB13 mutations in a population-based, case-control study of prostate cancer.', *The Prostate*. Wiley Online Library, 73(6), pp. 634–41. doi: 10.1002/pros.22604.

Witte, J. S. *et al.* (2013) 'HOXB13 mutation and prostate cancer: studies of siblings and aggressive disease.', *Cancer Epidemiology, Biomarkers & Prevention*. AACR, 22(4), pp. 675–80. doi: 10.1158/1055-9965.EPI-12-1154.

Xu, J. *et al.* (2013) 'HOXB13 is a susceptibility gene for prostate cancer: results from the International Consortium for Prostate Cancer Genetics (ICPCG).', *Human Genetics*. Springer, 132(1), pp. 5–14. doi: 10.1007/s00439-012-1229-4.

Supplementary Fig. 1 – Systematic review and meta-analysis: Flowchart detailing the identification of original research articles on the relative risk of prostate cancer for *HOXB13* G84E mutation carriers

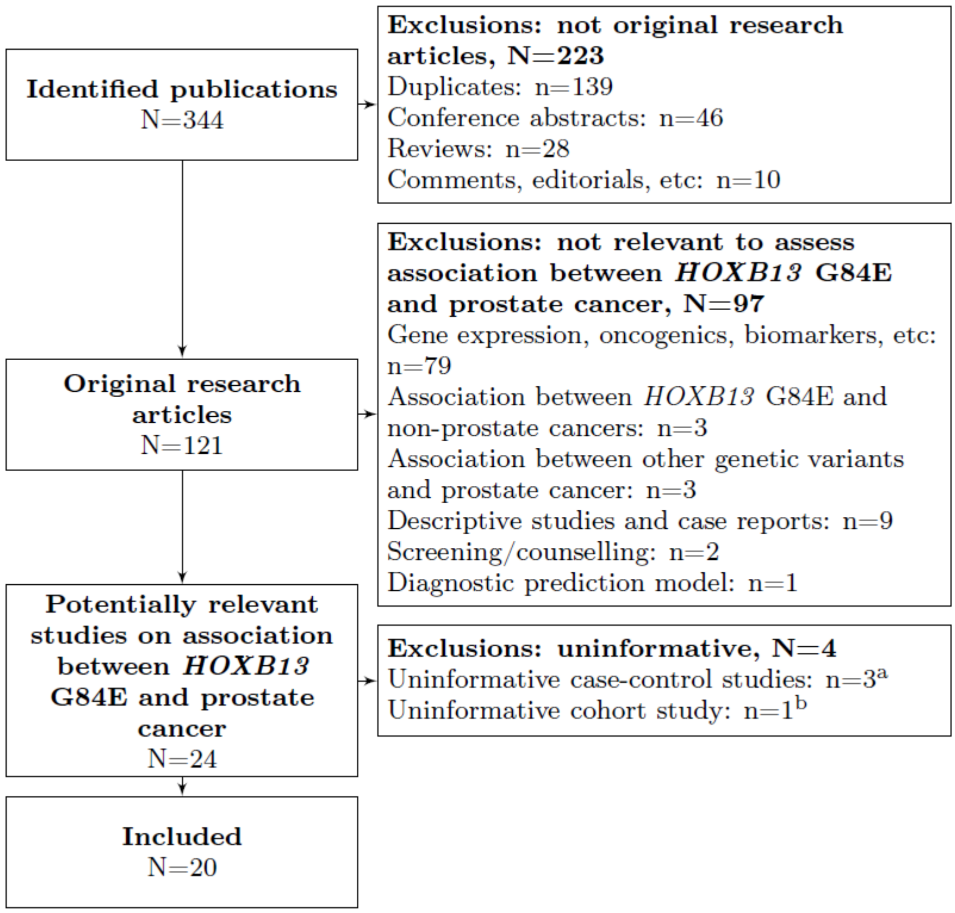

[a]No mutation carriers in cases and/or controls: n=2. Pooled analysis of previously published studies: n=1.
[b]No prostate cancers observed in mutation carriers: n=1.

Supplementary Table 1 – Systematic review and meta-analysis: Summary of published studies that estimate the relative risk of prostate cancer for *HOXB13* G84E mutation carriers

Case-control studies

Publication	Population	Dataset	Case selection	Control selection	Cases Mutation-carriers	Cases Total	Controls Mutation-carriers	Controls Total	Cases Average Age	Controls Average Age	RR	95% CI	Adjustments	Age-adjustment	Comment
Ewing *et al.*, 2012															
	USA, European ancestry	University of Michigan Prostate Cancer Genetics Project (UM-PCGP)	Young age (≤55 years) and/or familial (≥1 affected FDR or SDR)	Screening-negative[a]	26	1130	1	1401	Mean: 52.2, Median: 52	Not stated	33.0		No	No, but used screening-negative controls	
	USA, European ancestry	John Hopkins University	Mixed, unselected and familial (≥2 affected FDR)	Screening-negative[a]	46	3953	1	1401	Mean: 58.5, Median: 59	Not stated	16.5		No	No, but used screening-negative controls	
Akbari *et al.*, 2012															
	Canada, European ancestry	Sunnybrook	Unselected	Screening-negative	10	1525	2	1759	Mean: 65.6	Mean: 63.0	5.8	1.3-26.5	No	No, but used screening-negative controls	
Breyer *et al.*, 2012															
	USA, European ancestry	Familial Prostate Cancer Study	Familial (≥1 affected FDR or SDR)	Screening-negative[a]	16	858	2	825	Mean: 60.1	Mean: 60.1	7.9	1.8-34.5	Age	Covariate adjustment	
	USA, European ancestry	Singleton cases	Unselected	Screening-negative[a]	4	268	2	825	Mean: 58.4	Mean: 60.1	5.6	0.9-33.9	Age	Covariate adjustment	
Gudmundsson *et al.*, 2012															
	Iceland		Unselected	Other disease		4537		54444	Mean: 71	Mean: 84	3.67	1.71-7.90	No	No	Per allele OR; Relied on imputed *HOXB13* G84E status

	USA		Unselected	Population-based		1982		1260	Mean: 60, Median: 59	Not stated	14.7	3.59-60.14	No	No	Per allele OR
	Spain[b]		Unselected	Other disease		716		1692	Mean: 69, Median: 70	Not stated	∞		No	No	Per allele OR
	The Netherlands		Unselected	Population-based		1520		1916	64.3	Not stated, however age-matched to cases	7.51	3.99-14.11	No	Matching	Per allele OR
	Romania[c]		Unselected	Other disease		722		857	Mean: 70, Median: 71	Mean: 60, Median: 62	1.19	0-∞	No	No	Per allele OR
	UK	ProtecT	Young age (50-69 years)	Screening-negative		511		1825	Mean: 63.0	Mean: 62.5	14.44	4.74-44.03	No	No, but used screening-negative controls	Per allele OR
Kluźniak *et al.*, 2013															
	Poland		Unselected	Population-based	20	3515	3	2604	Mean: 68.8	Mean: 61.8	5	1.5-16.7	No	No	
Laitinen *et al.*, 2013[d]															
	Finland	Unselected cases vs Population controls	Unselected	Population-based[a]		3197		923	Mean: 68.6	Not stated	3.6	2.2-5.7	No	No	
	Finland	ERSPC screening cases vs ERSPC screening controls	Unselected	Screening-negative		1184		4544	Mean: 67.0	Not stated	2.1	1.2-3.6	No	No, but used screening-negative controls	
	Finland	Familial cases[e] vs Population controls	Familial (≥1 affected FDR or SDR)	Population-based[a]		114		923	Mean: 62.8	Not stated	8.2	4.3-16.0	No	No	
Stott-Miller *et al.*, 2013															

	USA, European ancestry	Fred Hutchinson Cancer Research Center (FHCRC)	Young age (two data collections: 40-64 years in first, 35-74 years in second)	Population-based	17	1310	5	1259	Mean: ~60.3; Not stated, but approxi-mated from age-group frequen-cies[f]	Mean: ~60.0; Not stated, but approxi-mated from age-group frequen-cies[f]	3.3	1.21-8.96	Age	Matching plus covariate adjustment	
Witte *et al.*, 2013															
	USA, mixed ancestry		Mixed, familial (all affected brothers from families with ≥1 brother diagnosed at age ≤73 years), or aggressive disease (Gleason score ≥7, stage ≥T2c, or PSA >10 ng/ml)	Mixed, family-based (healthy brothers) and unrelated (screening-negative)	20	1645	3	1019	Mean: 63.2	Mean: 64.3	4.79		Age, race and institution	Covariate adjustment	
Xu *et al.*, 2013															
	International	International Consortium for Prostate Cancer Genetics (ICPCG)[g]	Familial (affected men in multiple case families with at least one *HOXB13* G84E mutation carrier;	Family-based (unaffected men in multiple case families with at least one *HOXB13* G84E mutation	154	326	36	117	Mean: 64.4	Not stated	4.3	2.32-7.96	No	No	

			varying definitions)	carrier; varying definitions)											
Karlsson *et al.*, 2014															
	Sweden	Cancer of the Prostate Sweden (CAPS)	Unselected	Population-based	130	2805	24	1709	Median: 64.9	Median: 67.2	3.4	2.2-5.4	No	Matching	
	Sweden	Stockholm-1	Unselected	Screening-negative	91	2098	37	2880	Median: 65.8	Median: 64.0	3.5	2.4-5.2	No	No, but used screening-negative controls	
Albitar *et al.*, 2015															
	USA, ancestry not specified		Unselected	Other disease	2	232	1	110	Median: 68	Median: 65			No	No, but controls were of similar ages	
Beebe-Dimmer *et al.*, 2015															
	USA, mixed ancestry	Mayo Clinic Biobank	Unselected	Population-based	19	1362	23	5898	Not stated	Not stated	1.99	1.37-2.90	Age	Covariate adjustment	
Hoffmann *et al.*, 2015[h]															
	USA, European ancestry	Kaiser Permanente Research Program on Genes, Environment and Health Genetic Epidemiology Research on Adult Health and Aging (RPGEH GERA)	Unselected	Population-based		3976		29517	Not stated	Not stated	3.63	2.48-5.85	Age and genetic ancestry	Covariate adjustment	Relied on imputed *HOXB13* G84E status
Kote-Jarai *et al.*, 2015															
	UK	PRM probands vs controls	Unselected	Screening-negative[a]	38	3494	28	5252	Not stated	Mean: 55	2.12	1.30-3.46	No	No	

	UK	PRS probands vs controls	Familial (men from families with ≥2 affected FDR, SDR or TDR, one of whom diagnosed at age ≤65 years; or, ≥3 FDR, SDR or TDR diagnosed at any age)	Screening-negative[a]	25	786	28	5252	Not stated	Mean: 55	6.36	3.67-11.01	No	No	
	UK	PRY probands vs controls	Young age (≤60 years)	Screening-negative[a]	71	4373	28	5252	Not stated	Mean: 55	3.19	2.03-5.01	No	No	
Storebjerg *et al.*, 2016															
	Denmark	Aarhus Prostate Cancer Project	Unselected	Screening-negative	25	995	8	1622	Mean: 62.7, Median: 63	Mean: 61.2, Median: 62	5.12	2.26-13.38	No	No, but used screening-negative controls	
Karyadi *et al.*, 2017[i]															
	USA	PLCO	Unselected	Screening-negative	47	4222	9	2899	Mean: ~69.3; Not stated, but approxi-mated from age-group frequen-cies[f]	Mean: ~68.3; Not stated, but approxi-mated from age-group frequen-cies[f]	3.78	1.94-8.28	Age	Covariate adjustment	
FitzGerald *et al.*, 2017															
	Australia		Mixed, familial (affected men from	Mixed, family-based (unaffected		744		805	Not stated	Not stated	6.59[j]		No	No	

			families with ≥5 affected relatives) and young age (<75 years)	men from families with ≥5 affected relatives) and population-based											
Chen *et al.*, 2018															
	Norway	Norwegian Urological Cancer Group	Unselected	Population-based	18	779	12	1643	Not stated	Median: 64	3.8		No	No	

Other study designs

Publication	Population	Dataset	Inclusion criteria	Follow-up for PCa onset	Observed rates	RR	95% CI	Adjustments
Prospective cohort studies								
Chen *et al.*, 2013	International	REDUCE trial	Aged 50-75 years, PSA of 2.5-10 ng/ml if <60 years old or 3-10 ng/ml if ≥60 years old, prostate volume ≤80 cm^3, negative initial biopsy, no high-grade prostatic intraepithelial neoplasia or atypical small acinar proliferation	4 years	No. PCa/Total No. (%): 7/13 (54%) Mutation carriers 701/3186 (22%) Non-carriers	2.45	1.48-4.07	No
Laitinen *et al.*, 2013	Finland	BPH	Diagnosed with benign prostate hyperplasia (BPH)	8-14 years	254 PCa (2.6% mutation carriers) 516 without PCa (0.6% mutation carriers)	4.6	1.3-16.2	No
Kin-cohort study								
MacInnis *et al.*, 2013	Australia		Family members of men with PCa at age <55 years, with additional inclusion of		9 relatives with PCa, in 19 families with a mutation carrier proband	16.4	2.5-107.2	Ascertainment

			men diagnosed at ages 55-59 years some study years					

[a] Used as control group to multiple case series in same publication.
[b] Omitted from meta-analysis: no mutation carriers in controls.
[c] Omitted from meta-analysis: infinite confidence interval.
[d] Includes three case datasets (Unselected, Familial, and ERSPC) and two control datasets (Population, and EPSPC), and all combinations of case and control datasets are compared in the publication. For this meta-analysis, we selected the comparisons listed in the table so as to not count the same study populations multiple times.
[e] Restricted to familial cases not previously reported on in Xu *et al.* (2013).
[f] The mean age was approximated based on the frequencies and midpoint age of each age group.
[g] Excludes families previously reported on in Ewing *et al.* (2012), but includes 5 *HOXB13* G84E carrier families from the UKGPCS familial PRS cohort.
[h] Also analysed same data as a retrospective cohort and presented hazard ratios. This is not included in the present meta-analysis, to not count the same study populations multiple times.
[i] Karyadi *et al.* (2017) also reports results from the FHCRC study, which were previously published in Stott-Miller *et al.* (2013). Hence, we do not include the FHCRC results from Karyadi *et al.* (2017) in the present meta-analysis.
[j] Calculated by a quasi-likelihood based method which accounts for relatedness between subjects but does not produce confidence intervals.

Supplementary Fig. 2 – Systematic review and meta-analysis: Funnel plot of published relative risk estimates from unselected case-control studies

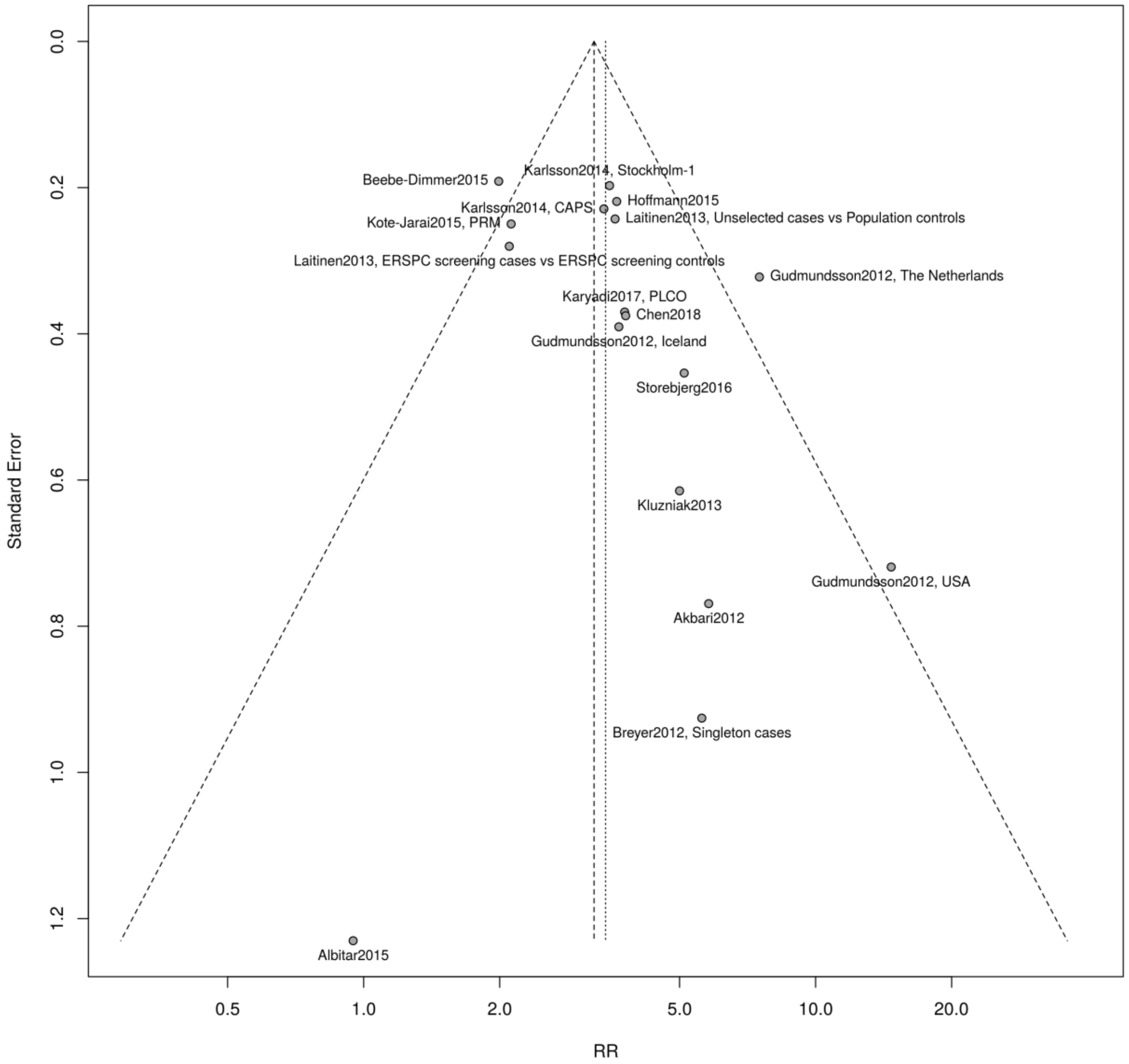

Supplementary Table 2 – Systematic review and meta-analysis: Leave-one-out sensitivity analysis for the unselected case-control studies

Omitted study	Fixed effects RR (95% CI)	Random effects RR (95% CI)	I^2, p-value
After omitting: Akbari2012	3.22 (2.78-3.72)	3.40 (2.75-4.21)	44%, p=0.029
After omitting: Breyer2012, Singleton cases	3.22 (2.79-3.73)	3.42 (2.76-4.23)	45%, p=0.027
After omitting: Gudmundsson2012, Iceland	3.22 (2.78-3.73)	3.43 (2.75-4.28)	45%, p=0.025
After omitting: Gudmundsson2012, USA	3.18 (2.75-3.68)	3.30 (2.72-4.02)	35%, p=0.083
After omitting: Gudmundsson2012, The Netherlands	3.09 (2.66-3.58)	3.18 (2.64-3.83)	26%, p=0.2
After omitting: Kluzniak2013	3.21 (2.78-3.72)	3.40 (2.74-4.22)	45%, p=0.028
After omitting: Laitinen2013, Unselected cases vs Population controls	3.20 (2.75-3.73)	3.44 (2.73-4.32)	45%, p=0.026
After omitting: Laitinen2013, ERSPC screening cases vs ERSPC screening controls	3.34 (2.87-3.88)	3.56 (2.88-4.42)	40%, p=0.050
After omitting: Karlsson2014, CAPS	3.22 (2.76-3.75)	3.46 (2.75-4.36)	45%, p=0.025
After omitting: Karlsson2014, Stockholm-1	3.19 (2.73-3.73)	3.45 (2.73-4.36)	45%, p=0.026
After omitting: Albitar2015	3.25 (2.81-3.76)	3.46 (2.81-4.27)	43%, p=0.033
After omitting: Beebe-Dimmer2015	3.52 (3.01-4.13)	3.60 (2.97-4.38)	25%, p=0.2
After omitting: Hoffmann2015	3.19 (2.73-3.72)	3.44 (2.73-4.33)	45%, p=0.027
After omitting: Kote-Jarai2015, PRM	3.37 (2.89-3.92)	3.57 (2.89-4.43)	39%, p=0.058
After omitting: Storebjerg2016	3.19 (2.76-3.70)	3.37 (2.72-4.18)	43%, p=0.033
After omitting: Karyadi2017, PLCO	3.21 (2.77-3.73)	3.42 (2.74-4.27)	45%, p=0.026
After omitting: Chen2018	3.21 (2.77-3.73)	3.42 (2.74-4.27)	45%, p=0.026

Supplementary Fig. 3 – Systematic review and meta-analysis: Funnel plot of published relative risk estimates from unselected case-control studies, after omission of Beebe-Dimer *et al*. (2015)

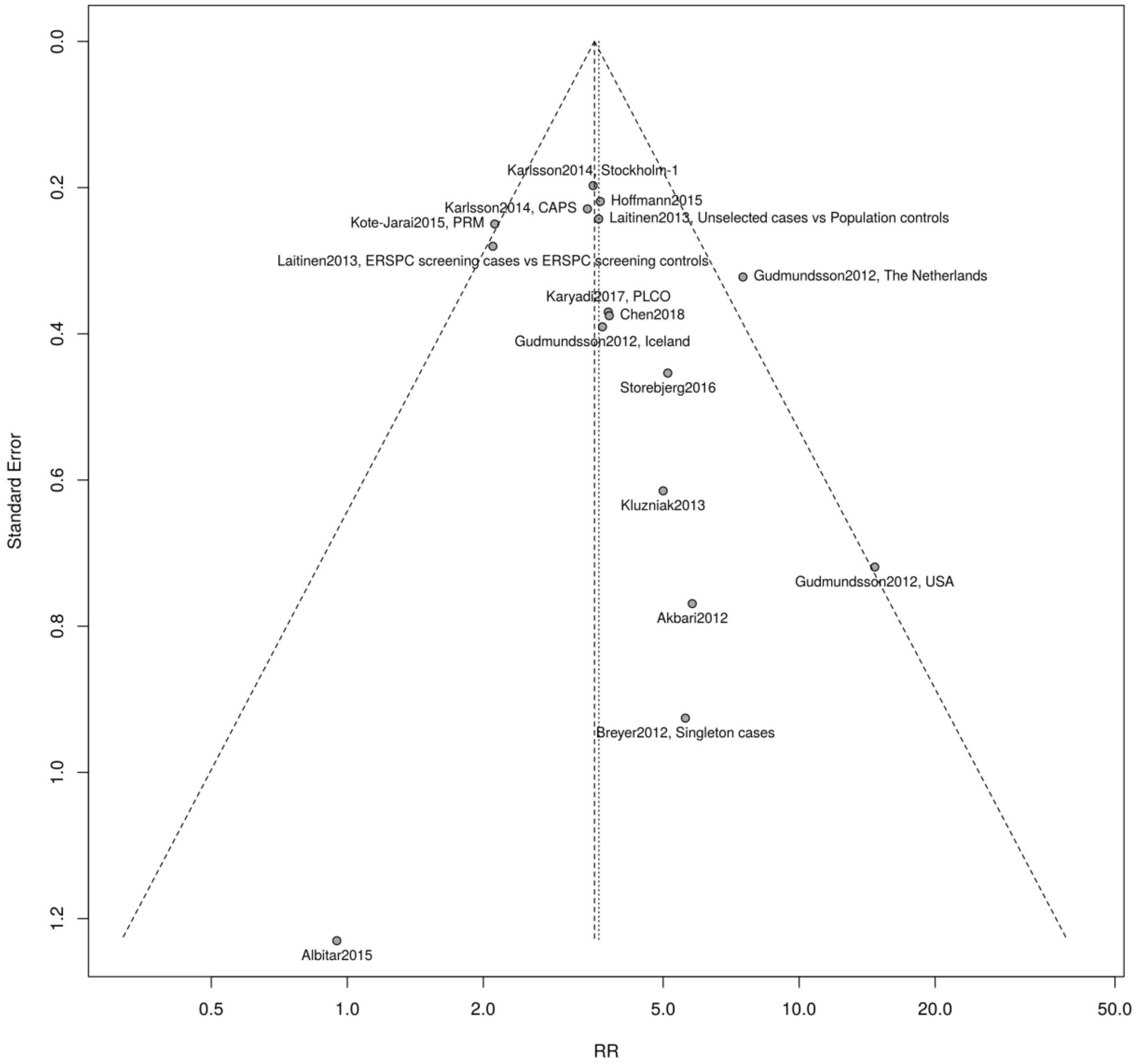

Model parameterisation

The PCa incidence was assumed to follow a model of the form

$$\lambda(t_i,k_i) = \lambda_0(t_i,k_i) \times \exp(G_i(t_i,k_i) + P_i)$$

where $\lambda_i(t_i,k_i)$ is the incidence for the ith man at age t_i in birth cohort k_i; $\lambda_0(t_i,k_i)$ is the baseline incidence; G_i is a variable dependent on the man's *HOXB13* G84E genotype and represents the RRs for heterozygous and/or homozygous mutation carriers (depending on the model of inheritance); and P_i is the man's polygenotype.

We constrained the average PCa incidence across all genotypes to agree with calendar-period- and cohort-specific PCa incidences for England and Wales (1,2). We split individuals into birth cohorts according to years of birth ≤1909, 1910-1919, ..., ≥1960, and assumed that the PCa incidence in each birth cohort followed that of those born in the mid-point years (e.g. 1915 for the second birth cohort, and using 1905 and 1965 for the earliest and latest cohort, respectively). To avoid large variations in incidences between successive years, we smoothed population incidences using LOWESS regression (3).

We parameterised the model in terms of the relative risk (RR) at the average polygenic load, i.e. averaged over the levels of the polygenic component in the population. Let $\overline{RR}_g(t,k)$ denote the RR at average polygenic load for men with g copies of *HOXB13* G84E compared to non-carriers. In this general form, $\overline{RR}_g$ may depend on age t and/or birth cohort k.

Assuming independence between *HOXB13* G84E and the polygenotype, and that the polygenotype acts multiplicatively with *HOXB13* G84E, the incidence at time t in birth cohort k for a man with *HOXB13* G84E genotype g and polygenotype P is

$$\lambda_{g,P}(t,k) = \lambda_{g,0}(t,k)e^P$$

where $\lambda_{g,0}(t,k)$ is the corresponding incidence when $P = 0$. The average incidence over the polygenotypes in the population is

$$\bar{\lambda}_g(t,k) = \frac{\sum_p \phi_p f_{g,p}(t,k)}{\sum_p \phi_p S_{g,p}(t-1,k)} = \frac{\sum_p \phi_p S_{g,p}(t-1,k)e^p}{\sum_p \phi_p S_{g,p}(t-1,k)} \lambda_{g,0}(t,k)$$

where ϕ_p is the population frequency of polygenotype p, $S_{g,p}(t-1,k)$ is the survival function for men with genotype g and polygenotype p, and $f_{g,p}(t,k) = \lambda_{g,p}(t,k)S_{g,p}(t-1,k)$ the corresponding failure density function.

Hence, the RR for mutation carriers with g copies of *HOXB13* G84E compared to non-carriers at average polygenic effect in the population is

$$\overline{RR}_g(t,k) = \frac{\bar{\lambda}_g(t,k)}{\bar{\lambda}_0(t,k)} = C_g(t,k)RR_{g,0}(t,k)$$

where $RR_{g,0}(t,k)$ is the RR at polygenotype $P = 0$ and

$$C_g(t,k) = \left(\frac{\sum_p \phi_p S_{g,p}(t-1,k)e^p}{\sum_p \phi_p S_{g,p}(t-1,k)}\right) \Big/ \left(\frac{\sum_p \phi_p S_{0,p}(t-1,k)e^p}{\sum_p \phi_p S_{0,p}(t-1,k)}\right).$$

Explicitly, the model given at the start of this appendix of the incidence for the *i*[th] individual with g_i copies of the *HOXB13* G84E mutation and polygenotype P_i, is thus specified with

$$G_i(t_i,k_i)=\begin{cases}\ln\left[C_g^{-1}(t_i,k_i)\overline{RR}_{g\geq 1}(t_i,k_i)\right]\times 1_{\{g_i\geq 1\}}, & \text{dominant model}\\ \ln\left[C_g^{-1}(t_i,k_i)\overline{RR}_{g=2}(t_i,k_i)\right]\times 1_{\{g_i=2\}}, & \text{recessive model}\\ \sum_{\gamma=1}^{2}\ln\left[C_g^{-1}(t,k_i)\overline{RR}_{\gamma}(t_i,k_i)\right]\times 1_{\{g_i=\gamma\}}, & \text{general model}\\ \ln\left[C_g^{-1}(t_i,k_i)\overline{RR}_{\text{per-allele}}(t_i,k_i)\right]\times g_i, & \text{multiplicative model}\end{cases}$$

where $1_{\{\ \}}$ is an indicator function taking values 0 or 1, and

$$P_i \sim N(0,\sigma_P^2)$$

is normally distributed with standard deviation σ_P.

In the main effects models, we assumed constant $\overline{RR}_g(t,k)\equiv\overline{RR}_g$. In addition, we assessed modification of the effect of G84E by allowing the RR to vary by age and birth cohort.

When exploring modification in the effect of *HOXB13* G84E between birth cohort groups, we divided men into N_C birth cohort groups and estimated separate RR as

$$\ln\overline{RR}_g(t,k)=\sum_{c=1}^{N_C}\beta_c\times 1_{\{k\,\in\,\text{group }c\}}$$

where β_c is the ln*RR* in the *c*[th] birth cohort group.

Similarly, we allowed the RR to vary between age groups by an analogue parameterisation. We also allowed the RR to vary with age according to a log-linear model as

$$\ln\overline{RR}_g(t,k)=\alpha_0+\alpha_1\times(t-70)$$

where α_0 corresponds to the estimated ln*RR* at age 70 and α_1 the change in ln*RR* per year of age.

Finally, we fit a model which allowed for modification by both age and birth cohort, by fitting separate birth cohort group specific intercepts in the above log-linear model as

$$\ln\overline{RR}_g(t,k)=\sum_{c=1}^{N_C}\gamma_c\times 1_{\{k\,\in\,\text{group }c\}}+\alpha\times(t-70)$$

where γ_c is the ln*RR* at age 70 in the *c*[th] birth cohort group, and α the change in ln*RR* per year of age.

References

1. Parkin DM, Ferlay J, Curado MP, Bray F, Edwards B, Shin HR, et al. Cancer incidence in five continents (CI5) volumes I to X, IARC CancerBase no. 12 [Database].
2. Office for National Statistics. Cancer registration statistics, England, 2010-2015 [Dataset].
3. Antoniou AC, Pharoah PD, McMullan G, Day NE, Ponder BA, Easton D. Evidence for further breast cancer susceptibility genes in addition to BRCA1 and BRCA2 in a population-based study. Genet Epidemiol [Internet]. 2001 Jul;21(1):1–18. Available from: http://www.ncbi.nlm.nih.gov/pubmed/11443730

All models

Supplementary Table 3 shows the main effects models for the *HOXB13* G84E mutation under different assumptions for the mode of inheritance. We found that a multiplicative model for the *HOXB13* G84E mutation fits the data best as measured by the Akaike information criterion (AIC), both for single gene models (AIC=44312.8) and models which allowed for a polygenic component to capture residual familial effects (AIC=40616.1). However, multiplicative, dominant and general models of inheritance showed similar model fit for both single gene models (multiplicative: AIC=44312.8; dominant: AIC=44314.9; general: AIC=44314.7) and polygenic models (polygenic multiplicative: AIC=40616.1; polygenic dominant: AIC=40619.4; polygenic general: AIC=40617.8).

Models which allowed for a polygenic component improved the AIC as compared to the corresponding single gene model for every assumed mode of inheritance for *HOXB13* G84E (polygenic multiplicative: AIC=40616.1, versus multiplicative: AIC=44312.8; polygenic dominant: AIC=40619.4, versus dominant: AIC=44314.9; polygenic recessive: AIC=40635.8, versus recessive: AIC=44368.2; polygenic general: AIC=40617.8, versus general: AIC=44314.7).

As the polygenic multiplicative model had the overall lowest AIC among the considered models, we chose the polygenic multiplicative model as the main model for all subsequent analyses.

Supplementary Table 3 – All models: Main effects models by assumed inheritance

**Model**	**Log-likelihood**	**No. parameters**	**AIC[a]**	**Likelihood ratio test p-value**	***HOXB13* G84E**	**RR[b]**	**(95% CI[c])**	**Minor allele frequency**	**(95% CI)**	**Polygenic standard deviation**	**(95% CI)**
Sporadic	-22182.9	1	44367.8	-		1.00[d]		0.77%	(0.67%-0.89%)	0.00[d]	
Dominant	-22155.5	2	44314.9	<0.001[e]	Het/Hom[f]	3.84	(3.02-4.89)	0.17%	(0.13%-0.24%)	0.00[d]	
Recessive	-22182.1	2	44368.2	0.2[e]	Hom	2.81	(0.61-12.9)	0.76%	(0.66%-0.88%)	0.00[d]	
General	-22154.4	3	44314.7	<0.001[e]	Het	3.81	(2.99-4.86)	0.17%	(0.13%-0.24%)	0.00[d]	
					Hom	12.9	(3.40-49.0)				
Multiplicative	-22154.4	2	44312.8	<0.001[e]	Per-allele	3.79	(3.00-4.80)	0.18%	(0.13%-0.24%)	0.00[d]	
Polygenic	-20315.6	2	40635.2	<0.001[e]				0.77%	(0.67%-0.89%)	2.73	(2.65-2.81)
Polygenic dominant	-20306.7	3	40619.4	<0.001[g]	Het/Hom	3.52	(1.78-6.97)	0.22%	(0.11%-0.44%)	2.72	(2.64-2.80)

Polygenic recessive	-20314.9	3	40635.8	0.2[g]	Hom	2.70	(1.43-5.08)	0.76%	(0.66%-0.88%)	2.73	(2.65-2.81)
Polygenic general	-20304.9	4	40617.8	<0.001[g]	Het	3.56	(1.72-7.34)	0.22%	(0.11%-0.45%)	2.72	(2.64-2.80)
					Hom	19.5	(3.24-117.6)				
Polygenic multiplicative	-20305.0	3	40616.1	<0.001[g]	Per-allele	3.86	(2.16-6.88)	0.20%	(0.11%-0.36%)	2.72	(2.64-2.80)

[a] AIC: Akaike information criterion.
[b] RR: Relative risk.
[c] CI: Confidence interval.
[d] Constrained to constant value.
[e] Compared to sporadic model.
[f] Het: heterozygous carriers; Hom: homozygous carriers.
[g] Compared to polygenic model.

Supplementary Table 4 shows the polygenic multiplicative model refitted with alternative assumptions to those used for the main model.

The main polygenic multiplicative model assumes calendar-period- and birth-cohort-specific population incidences.

- When we instead assumed that the latest available (2015) population incidence applied to all men, the model fit was considerably worse (AIC=83526.5, versus AIC=40616.1 for the main model).

The main polygenic multiplicative model assumes the polygenic component to act multiplicatively on both mutation carriers and non-carriers with equal polygenic standard deviation.

- When we assumed that the polygenic component does not act on *HOXB13* G84E mutation carriers (i.e. that the polygenic standard deviation is equal to zero for mutation carriers), the model fit was worse (AIC=40666.4, versus AIC=40616.1 for the main model).
- When we allowed for the polygenic component to act differently on *HOXB13* G84E mutation carriers and non-carriers (i.e. estimating separate polygenic standard deviations for mutation carriers and non-carriers), the AIC did not improve (AIC=40616.8, versus AIC=40616.1 for the main model), and there was no statistically significant difference between this model and the main model (p=0.3). There was a statistically significant difference between the model which allowed a polygenic standard deviation for mutation carriers, and the model where the polygenic component did not act on mutation carriers (p<0.001).

As these results support the initial assumptions of calendar-period- and birth-cohort-specific population incidences, as well as of a polygenic component that acts multiplicatively on both mutation carriers and non-carriers, we chose to retain the main polygenic multiplicative model.

Supplementary Table 4 – All models: Polygenic multiplicative model, alternative fits

Model	Log-likelihood	No. parameters	AIC[a]	Likelihood ratio test p-value	Group	Per-allele RR[b]	(95% CI[c])	Minor allele frequency	(95% CI)	Polygenic standard deviation	(95% CI)
Assuming latest available population incidence (2015) for all birth cohorts	-41760.2	3	83526.5	-		4.20	(3.49-5.04)	0.14%	(0.10%-0.18%)	3.26	(3.23-3.30)
Assuming polygenic component does not act on mutation carriers	-20330.2	3	40666.4	-		9.73	(7.74-12.2)	0.09%	(0.07%-0.11%)		
					Non-carriers					2.72	(2.64-2.80)
					G84E carriers					0.00[d]	
Assuming separate polygenic standard	-20304.4	4	40616.8	0.3[e]; <0.001[f]		4.98	(2.74-9.05)	0.16%	(0.09%-0.29%)		

deviation for mutation carriers	Non-carriers	2.72	(2.64-2.80)
	G84E carriers	3.28	(2.63-4.09)

[a] AIC: Akaike information criterion.
[b] RR: Relative risk.
[c] CI: Confidence interval.
[d] Constrained to constant value.
[e] Compared to polygenic multiplicative model.
[f] Compared to polygenic multiplicative model where the polygenic component does not act on mutation carriers.

Supplementary Table 5 shows the considered polygenic multiplicative models that allowed for separate RRs for subgroups of *HOXB13* G84E mutation carriers split by age and/or birth cohort.

As compared to the main polygenic multiplicative model, a model which allowed the RR for *HOXB13* G84E mutation carriers to vary by ten-year age groups did not improve the fit (AIC=40621.0, versus AIC=40616.1 for the main model; p=0.5), but point estimates indicated higher risks at younger ages. Most age-specific RR models with broader age groups similarly showed no improvement in model fit (four age groups: AIC=40619.4; three age groups: AIC=40619.3). Only one of the considered models with dichotomous age groups showed a minor improvement in AIC (ages 35-54 and 55-84: AIC=40615.6, versus AIC=40616.1 for the main model; p=0.12). The best fitting age-specific model was one which allowed the log-RR to vary linearly with age (AIC=40614.8, versus AIC=40616.1 for the main model; p=0.068).

A model with separate RRs for *HOXB13* G84E mutation carriers for each of the seven birth cohorts showed improved fit over the main polygenic multiplicative model (AIC=40612.1, versus AIC=40616.1 for the main model; p=0.014). When we created broader birth cohort groups by collapsing adjacent birth cohorts, results were similar (four birth cohort groups: AIC=40613.1; three birth cohort groups: AIC=40613.4). The most parsimonious cohort-specific model as measured by AIC, was a model with two birth cohort groups split by years-of-birth ≤1929 and ≥1930 (AIC=40609.6, versus AIC=40616.1 for the main model; p=0.004).

The best fitting model with birth-cohort-specific RRs fit the data better than the best fitting model with age-specific RRs (AIC=40609.6 for the model with two birth-cohort-specific RRs, versus AIC=40614.8 for the model where the RR varied log-linearly with age). A model that allowed the RR for *HOXB13* G84E mutation carriers to vary by both age and birth cohort, according to the parametrisations used in the best fitting age- and cohort-specific models, did not improve the model fit (AIC=40611.3, versus AIC=40609.6 for the most parsimonious model with birth-cohort-specific RRs; p=0.6). In this model with age- and birth-cohort-specific RRs, the point estimates for the birth-cohort-specific RRs were similar to those of the birth-cohort-specific model (≤1929: RR=3.09 versus RR=3.13; ≥1930: RR=5.96 versus RR=5.71), but the age-specific effect was attenuated as compared to the age-specific model (per-year-of-age RR=1.00, versus per-year-of-age RR=0.98).

We thus chose the model with the two birth cohort groups ≤1929 and ≥1930 as the most parsimonious model out of all considered.

Supplementary Table 5 – All models: Polygenic multiplicative model, RR modified by age and/or birth cohort

Model	Log-likelihood	No. parameters	AIC[a]	Likelihood ratio test p-value	Group	Per-allele RR[b]	(95% CI[c])	Minor allele frequency	(95% CI)	Polygenic standard deviation	(95% CI)
Age-specific RR: five age groups	-20303.5	7	40621.0	0.5[d]				0.19%	(0.12%-0.31%)	2.72	(2.64-2.80)
					Age 35-44	7.06	(2.12-23.5)				
					Age 45-54	4.83	(2.67-8.73)				
					Age 55-64	3.91	(2.37-6.43)				
					Age 65-74	3.73	(2.31-6.02)				

					Age 75-84	3.46	(1.94-6.16)				
Age-specific RR: four age groups	-20303.7	6	40619.4	0.4[d]				0.19%	(0.11%-0.33%)	2.72	(2.64-2.80)
					Age 35-54	4.99	(2.81-8.85)				
					Age 55-64	3.95	(2.25-6.92)				
					Age 65-74	3.77	(2.23-6.35)				
					Age 75-84	3.49	(1.57-7.77)				
Age-specific RR: three age groups	-20304.6	5	40619.3	0.7[d]				0.19%	(0.12%-0.30%)	2.72	(2.64-2.80)
					Age 35-64	4.22	(2.63-6.77)				
					Age 65-74	3.76	(2.38-5.93)				
					Age 75-84	3.49	(1.88-6.50)				
Age-specific RR: dichotomous age groups	-20304.6	4	40617.1	0.3[d]				0.20%	(0.12%-0.34%)	2.72	(2.64-2.80)
					Age 35-44	6.73	(2.62-17.3)				
					Age 45-84	3.79	(2.26-6.35)				
	-20303.8	4	40615.6	0.12[d]				0.20%	(0.12%-0.32%)	2.72	(2.64-2.80)
					Age 35-54	4.87	(2.90-8.18)				
					Age 55-84	3.77	(2.33-6.10)				
	-20304.7	4	40617.3	0.4[d]				0.19%	(0.11%-0.33%)	2.72	(2.64-2.80)
					Age 35-64	4.22	(2.39-7.45)				
					Age 65-84	3.70	(2.16-6.35)				
	-20304.9	4	40617.8	0.6[d]				0.20%	(0.11%-0.36%)	2.72	(2.64-2.80)
					Age 35-74	3.92	(2.20-7.01)				
					Age 75-84	3.41	(1.70-6.84)				
	-20303.4	4	40614.8	0.068[d]				0.18%	(0.11%-0.31%)	2.72	(2.64-2.80)
					Baseline (Age 70)	3.70	(2.23-6.14)				

Age-specific RR: per year of age (log-linear model)[e]					Per year of age	0.98	(0.97-1.00)				
Birth-cohort-specific RR: seven birth cohorts	-20297.1	9	40612.1	0.014[d]				0.13%	(0.08%-0.20%)	2.72	(2.64-2.80)
					Born ≤1909	1.59	(0.65-3.89)				
					Born 1910-19	4.64	(1.74-12.4)				
					Born 1920-29	3.64	(1.83-7.24)				
					Born 1930-39	6.32	(4.03-9.91)				
					Born 1940-49	6.08	(3.78-9.79)				
					Born 1950-59	6.37	(3.73-10.9)				
					Born ≥1960	11.8	(5.99-23.1)				
Birth-cohort-specific RR: four birth cohort groups	-20300.6	6	40613.1	0.030[d]				0.13%	(0.09%-0.21%)	2.72	(2.65-2.80)
					Born ≤1919	2.74	(1.54-4.87)				
					Born 1920-29	3.59	(1.94-6.65)				
					Born 1930-39	6.10	(3.89-9.58)				
					Born ≥1940	6.18	(3.86-9.89)				
Birth-cohort-specific RR: three birth cohort groups	-20301.7	5	40613.4	0.035[d]				0.12%	(0.08%-0.19%)	2.72	(2.65-2.80)
					Born ≤1919	2.80	(1.72-4.56)				
					Born 1920-39	5.69	(3.87-8.37)				
					Born ≥1940	6.62	(4.33-10.1)				
Birth-cohort-specific RR: dichotomous birth cohort groups	-20301.5	4	40611.0	0.008[d]				0.13%	(0.09%-0.19%)	2.72	(2.65-2.80)
					Born ≤1909	1.78	(0.77-4.11)				
					Born ≥1910	5.92	(4.11-8.52)				
	-20302.1	4	40612.3	0.016[d]				0.13%	(0.09%-0.19%)	2.72	(2.65-2.80)
					Born ≤1919	2.75	(1.63-4.63)				

					Born ≥1920	5.97	(4.17-8.54)				
	-20300.8	4	40609.6	0.004[d]				0.14%	(0.09%-0.21%)	2.72	(2.65-2.80)
					Born ≤1929	3.09	(2.03-4.71)				
					Born ≥1930	5.96	(4.01-8.88)				
	-20303.9	4	40615.8	0.13[d]				0.17%	(0.10%-0.31%)	2.72	(2.64-2.80)
					Born ≤1939	3.76	(2.19-6.45)				
					Born ≥1940	4.82	(2.61-8.91)				
	-20304.2	4	40616.5	0.2[d]				0.19%	(0.11%-0.32%)	2.72	(2.64-2.80)
					Born ≤1949	3.93	(2.36-6.52)				
					Born ≥1950	4.76	(2.60-8.72)				
	-20302.4	4	40612.7	0.021[d]				0.19%	(0.12%-0.33%)	2.72	(2.64-2.80)
					Born ≤1959	3.87	(2.32-6.45)				
					Born ≥1960	7.69	(3.75-15.8)				
Age- and birth-cohort-specific RR[f]	-20300.7	5	40611.3	0.020[g]; 0.6[h]				0.14%	(0.09%-0.21%)	2.72	(2.65-2.80)
					Born ≤1929 (Age 70)	3.13	(1.96-5.01)				
					Born ≥1930 (Age 70)	5.71	(3.71-8.78)				
					Per year of age	1.00	(0.98-1.01)				

[a] AIC: Akaike information criterion.
[b] RR: Relative risk.
[c] CI: Confidence interval.
[d] Compared to polygenic multiplicative model.
[e] The model with log-linear age-specific RR was specified as $\ln \overline{RR}\,(t) = \alpha_0 + \alpha_1 \times (t-70)$ at age t, where α_0 corresponds to the estimated RR at age 70 and α_1 the change in RR per year of age; see the Supplementary material (model parameterisation).
[f] The model with age- and birth-cohort-specific RR was specified as $\ln \overline{RR}\,(t,k) = \gamma_{\leq 1929} \times 1_{\{k\, \in \text{ birth cohort group } \leq 1929\}} + \gamma_{\geq 1930} \times 1_{\{k\, \in \text{ birth cohort group } \geq 1930\}} + \alpha \times (t-70)$ for birth cohort k at age t, where $\gamma_{\leq 1929}$ and $\gamma_{\geq 1930}$ corresponds to the estimated RR at age 70 for men born ≤1929 and ≥1930, respectively, and α the change in RR per year of age; see the Supplementary material (model parameterisation).

[g] Compared to polygenic multiplicative model with log-linear age-specific RR.
[h] Compared to polygenic multiplicative model RR specific to birth cohorts ≤1929/≥1930.

Supplementary Fig. 4 – Predicted cumulative prostate cancer risks for a 35 year old man carrying a single copy of the *HOXB13* G84E mutation with average (unknown) prostate cancer family history, as estimated by the most parsimonious model, by birth cohort

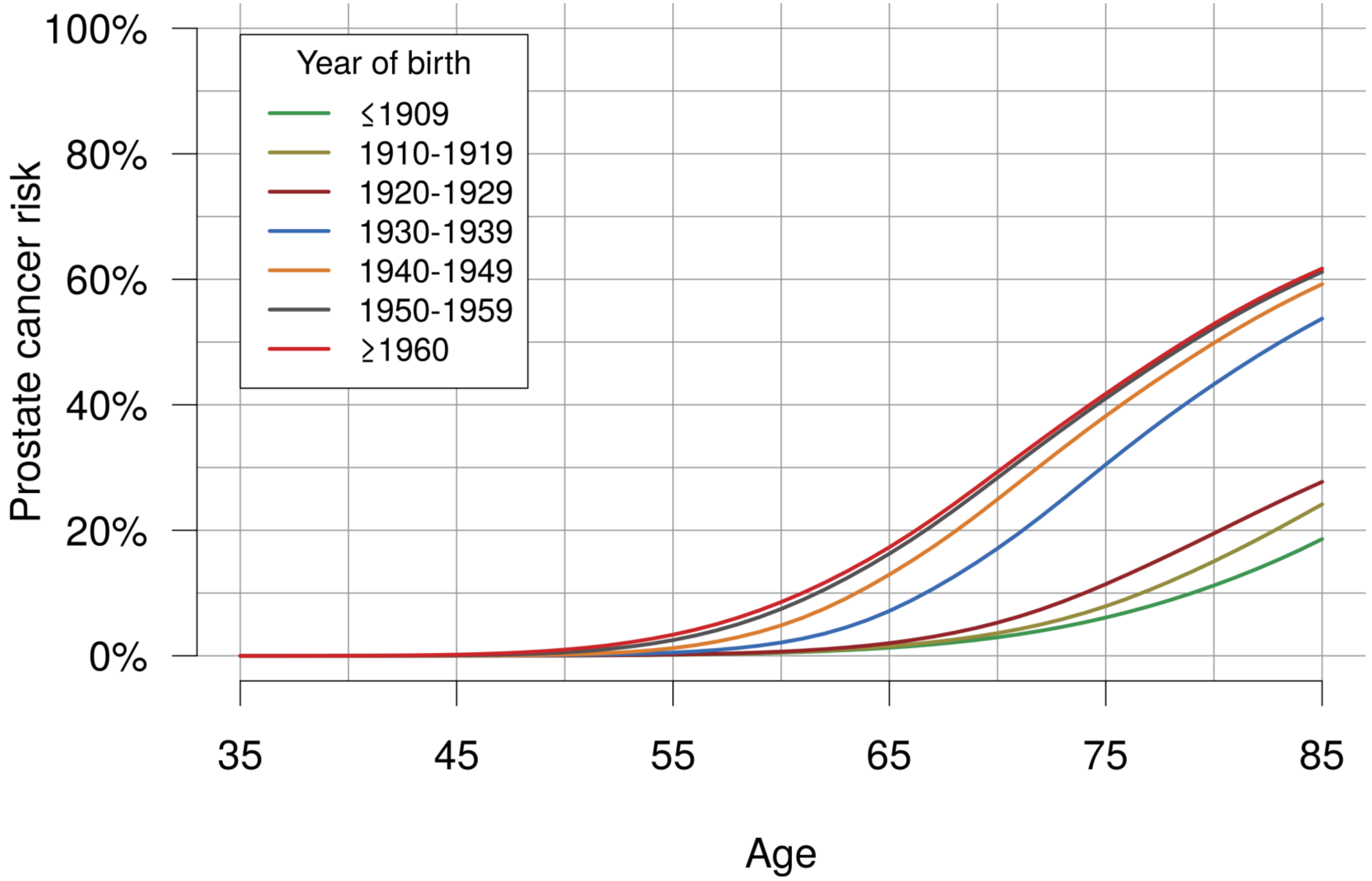

Sensitivity analyses

Supplementary Table 6 shows the results of the sensitivity analyses by refitting the most parsimonious model (with birth-cohort-specific RRs) to various subgroups of the data.

To assess the potential impact of self-reported family history on second-degree relatives, we refitted the most parsimonious model using family data on first-degree relatives only. The RR and minor allele frequency point estimates of the resulting model were similar to those of the model fitted to the entire dataset.

To assess the potential impact of imperfect ascertainment adjustments, we refitted the model to the population ascertained PRM and young-onset ascertained PRY arms separately. Refitting to the family ascertained PRS arm is not possible, because the residual familial polygenic component is not identifiable in this dataset due to that the ascertainment adjustment involves conditioning on all phenotypes in the relatives. The PRM arm yielded lower RR point estimates than the PRY arm (≤1929: RR=3.18 for PRM versus RR=4.01 for PRY; ≥1930: RR=4.72 for PRM versus RR=10.4 for PRY), but the confidence intervals were overlapping.

Supplementary Table 6 – Sensitivity analyses: Most parsimonious polygenic multiplicative model with birth-cohort-specific RRs, refitted to subgroups

Subgroup	Log-likelihood	No. parameters	AIC[a]	Likelihood ratio test p-value	Group	Per-allele RR[b]	(95% CI[c])	Minor allele frequency	(95% CI)	Polygenic standard deviation	(95% CI)
Subgroup: First degree-relatives[d]	-12708.0	4	25424.0	0.021[e]				0.13%	(0.08%-0.20%)	2.92	(2.85-3.00)
					Born ≤1929	3.54	(1.99-6.32)				
					Born ≥1930	6.31	(4.12-9.66)				
Subgroup: PRM (population ascertained)	-5000.1	4	10008.3	0.3[f]				0.14%	(0.05%-0.37%)	2.35	(2.16-2.54)
					Born ≤1929	3.18	(1.18-8.55)				
					Born ≥1930	4.72	(1.59-14.0)				
Subgroup: PRY (ascertained by young age at diagnosis)	-15164.6	4	30337.3	0.037[g]				0.08%	(0.04%-0.17%)	2.80	(2.72-2.89)
					Born ≤1929	4.01	(2.10-7.64)				
					Born ≥1930	10.4	(5.03-21.5)				

[a] AIC: Akaike information criterion.

[b] RR: Relative risk.
[c] CI: Confidence interval.
[d] First degree relatives for PRM and PRY cohorts, but first- and second-degree relatives for the PRS cohort where families were ascertained on the basis of family history.
[e] Compared to Polygenic multiplicative model, fit using first-degree relatives (not shown).
[f] Compared to Polygenic multiplicative model, fit using PRM families (not shown).
[g] Compared to Polygenic multiplicative model, fit using PRY families (not shown).

Supplementary Table 7 shows the results for the sensitivity analyses by refitting the most parsimonious model (with birth-cohort-specific RRs), to datasets where alternative imputation or censoring schemes had been used to handle missing data on ages-at-diagnosis. The primary dataset used in the analysis was based on sampling from the observed age-at-diagnosis distribution for men in the same birth cohort as those in the population-based PRM arm.

- To assess the potential impact of relying on the observed distribution in the population-based arm, we performed an alternative imputation which was based on external population-based data. We sampled ages-at-diagnosis from historical calendar-period- and birth-cohort-specific population prostate cancer incidences, adjusted for year-of-birth-specific mortality. The resulting point estimates were very similar to those from the primary dataset.
- To allow for the fact that ages-at-diagnosis in relatives of men with familial or young-age prostate cancer may differ from population distributions, we performed an alternative imputation based on the mean observed age-at-diagnoses within each ascertainment group and birth cohort. The resulting point estimates were very similar to those from the primary dataset.
- To assess the robustness of the results to extreme values, we performed three constant value imputations: assigning ages-at-diagnosis 60, 70 or 80 to all with missing ages-at-diagnosis. RR estimates were higher when age 60 was assumed and lower when age 80 was assumed, but all three imputations resulted in models which showed statistically significant differences in the RRs between the two birth cohort groups.
- To assess the impact of not performing imputations we instead censored all men with missing ages-at-diagnosis at age zero, which is conceptually equivalent to excluding all with missing ages-at-diagnosis. The resulting model showed overall lower RR point estimates, but the differences in RRs between the two birth cohort groups were statistically significant.
- To assess the impact if all men with missing ages-at-diagnoses had been diagnosed with prostate cancer at a high age, we assumed all with missing data to be censored at age 85. The resulting model showed lower RR point estimates, but the differences in RRs between the two birth cohort groups were statistically significant.

In summary, all alternative imputation and censoring schemes resulted in statistically significant differences in RRs between the two birth cohort groups based on years-of-birth ≤1929 and ≥1930.

Supplementary Table 7 – Sensitivity analyses: Most parsimonious polygenic multiplicative model with birth-cohort-specific RRs, alternative imputation schemes

**Imputation or censoring scheme**	**Log-likelihood**	**No. parameters**	**AIC[a]**	**Likelihood ratio test p-value**	**Group**	**Per-allele RR[b]**	**(95% CI[c])**	**Minor allele frequency**	**(95% CI)**	**Polygenic standard deviation**	**(95% CI)**
Alternative imputation: Sampled from historical population prostate cancer incidences[d]	-20316.4	4	40640.9	0.004[e]				0.14%	(0.09%-0.22%)	2.72	(2.65-2.80)
					Born ≤1929	3.01	(1.95-4.64)				
					Born ≥1930	5.80	(3.75-8.97)				
	-20393.4	4	40794.8	0.005[e]				0.14%	(0.09%-0.20%)	2.76	(2.69-2.84)

Alternative imputation: Mean observed age in each ascertainment group and birth cohort					Born ≤1929	3.15	(2.07-4.81)				
					Born ≥1930	6.06	(4.11-8.93)				
Alternative imputation: Assuming age 60 for all with unknown age at diagnosis	-24147.8	4	48303.6	0.046[e]				0.13%	(0.08%-0.20%)	2.95	(2.88-3.01)
					Born ≤1929	3.92	(2.22-6.93)				
					Born ≥1930	6.28	(4.06-9.72)				
Alternative imputation: Assuming age 70 for all with unknown age at diagnosis	-21356.7	4	42721.4	0.015[e]				0.12%	(0.08%-0.20%)	2.81	(2.74-2.88)
					Born ≤1929	4.02	(1.93-8.37)				
					Born ≥1930	6.70	(4.12-10.9)				
Alternative imputation: Assuming age 80 for all with unknown age at diagnosis	-20375.7	4	40759.3	0.017[e]				0.20%	(0.14%-0.30%)	2.59	(2.51-2.69)
					Born ≤1929	2.45	(1.70-3.53)				
					Born ≥1930	4.04	(2.75-5.93)				
Censoring all with unknown age at diagnosis at age 0	-11885.4	4	23778.8	0.013[e]				0.20%	(0.12%-0.33%)	1.70	(1.60-1.80)
					Born ≤1929	2.18	(1.32-3.60)				
					Born ≥1930	4.08	(2.48-6.70)				
Censoring all with unknown age at diagnosis at age 85	-12258.0	4	24524.0	0.014[e]				0.23%	(0.14%-0.38%)	1.30	(1.21-1.39)
					Born ≤1929	1.88	(1.17-3.02)				

(assuming diagnoses at ages above 85)	Born ≥1930	3.47	(2.15-5.59)

[a] AIC: Akaike information criterion.
[b] RR: Relative risk.
[c] CI: Confidence interval.
[d] Based on population prostate cancer incidences for England and Wales (1960-2015), adjusted for population mortality.
[e] Compared to the corresponding Polygenic multiplicative model, based on the data using the same alternative imputation or censoring scheme (not shown).
